# Using an autologistic regression model to identify spatial risk factors and spatial risk patterns of hand, foot and mouth disease (HFMD) in Mainland China

**DOI:** 10.1186/1471-2458-14-358

**Published:** 2014-04-14

**Authors:** Yan-Chen Bo, Chao Song, Jin-Feng Wang, Xiao-Wen Li

**Affiliations:** 1 State Key Laboratory of Remote Sensing Science, Research Center for Remote Sensing and GIS, and School of Geography, Beijing Normal University, Beijing 100875, China; 2 Beijing Key Laboratory of Environmental Remote Sensing and Digital City, Beijing 100875, China; 3 LREIS, Institute of Geographic Sciences and Natural Resources Research, Chinese Academy of Sciences, Beijing 100101, China; 4 Key Laboratory of Surveillance and Early-warning on Infectious Disease, Chinese Center for Disease Control and Prevention, Beijing 102206, China

## Abstract

**Background:**

There have been large-scale outbreaks of hand, foot and mouth disease (HFMD) in Mainland China over the last decade. These events varied greatly across the country. It is necessary to identify the spatial risk factors and spatial distribution patterns of HFMD for public health control and prevention. Climate risk factors associated with HFMD occurrence have been recognized. However, few studies discussed the socio-economic determinants of HFMD risk at a space scale.

**Methods:**

HFMD records in Mainland China in May 2008 were collected. Both climate and socio-economic factors were selected as potential risk exposures of HFMD. Odds ratio (OR) was used to identify the spatial risk factors. A spatial autologistic regression model was employed to get OR values of each exposures and model the spatial distribution patterns of HFMD risk.

**Results:**

Results showed that both climate and socio-economic variables were spatial risk factors for HFMD transmission in Mainland China. The statistically significant risk factors are monthly average precipitation (OR = 1.4354), monthly average temperature (OR = 1.379), monthly average wind speed (OR = 1.186), the number of industrial enterprises above designated size (OR = 17.699), the population density (OR = 1.953), and the proportion of student population (OR = 1.286). The spatial autologistic regression model has a good goodness of fit (ROC = 0.817) and prediction accuracy (Correct ratio = 78.45%) of HFMD occurrence. The autologistic regression model also reduces the contribution of the residual term in the ordinary logistic regression model significantly, from 17.25 to 1.25 for the odds ratio. Based on the prediction results of the spatial model, we obtained a map of the probability of HFMD occurrence that shows the spatial distribution pattern and local epidemic risk over Mainland China.

**Conclusions:**

The autologistic regression model was used to identify spatial risk factors and model spatial risk patterns of HFMD. HFMD occurrences were found to be spatially heterogeneous over the Mainland China, which is related to both the climate and socio-economic variables. The combination of socio-economic and climate exposures can explain the HFMD occurrences more comprehensively and objectively than those with only climate exposures. The modeled probability of HFMD occurrence at the county level reveals not only the spatial trends, but also the local details of epidemic risk, even in the regions where there were no HFMD case records.

## Background

Enterovirus 71 (EV71) is a common cause of hand, foot, and mouth disease (HFMD), may also cause severe neurological diseases, such as encephalitis and poliomyelitis-like paralysis. Outbreaks of hand, foot and mouth disease associated with EV71 infections have occurred in the Asia Pacific region since 1997 [1]. HFMD is a children's common infectious disease that mainly occurs in children under five years old [2]. In most cases, the disease is mild and self-limiting. However, severe clinical presentations with neurological symptoms such as meningitis, encephalitis, polio-like paralysis, and pulmonary edema may occur [3], which can cause serious injury or even death to young children. Outbreaks of HFMD have been reported many times in the countries of Western Pacific Region over the last decade [4-7]. There were large-scale HFMD outbreaks in Mainland China in 2008 and 2009 that led to 488,955 reported cases and a morbidity of 37/100,000 during the year 2008 and 1,155,525 cases, a mortality of 0.0095/100,000 and a fatality of 0.26/1000 during the year 2009 [2]. HFMD can produce a pandemic in a short period of time due to its highly infectious characteristic, thus poses a serious threat to the public health.

Many studies have extensively investigated the disease transmission characteristics, the risk factors, and the spatial distribution patterns of HFMD risk. It was found that the transmission pattern of HFMD shows strong seasonal characteristics. The epidemic peaks occurred in spring and early summer in Mainland China [2]. The highest incidence of HFMD was in summer in Taiwan, China [4]. The epidemics have inter-years periodic characteristics, as they occur once every three years In Malaysia [8]. At the temporal scale of weeks, HFMD incidence has a significant association with weekly temperature and precipitation with 1–2 weeks in Singapore [9] and 7 weeks in China [10].

Odds ratio (OR) is used to measure the risk of a disease exposed to the determinants, which has been widely used to identify risk factors in epidemiology [11]. It indicates the strength of the association between the exposures and disease. An OR value greater than 1 indicates that the exposure is a risk factor, a value less than 1 indicates a protective factor and a value equal to 1 indicates an unrelated factor. Previous studies on the OR values of the significant HFMD risk factors showed that 1.0 to 2.9 years old children have the highest risk (OR > 2.3). Boys were more susceptible than girls (OR > 1.56). Infant cases had the highest incidences of severe disease (OR > 1.4) and death (OR > 2.4). Enterovirus 71 is more strongly associated with severe disease compared with Coxsackie A16 (OR > 16) [12]. Playing with neighborhood children (OR = 11), visiting an outpatient clinic for another reason > 1 week before the onset of HFMD (OR = 20), community exposures to crowded places (OR = 7.3) [13], rural/urban areas (OR = 2.1), drinking behavior (OR = 2.441), infant hand washing before/after dinner (OR = 0.505) [14], float population (OR = 4.507), toy sucking (OR = 3.220) [15] and being in a low-income families are other risk factors [16].

Besides the personal characteristics above, climate variables as the spatial risk factors associated with HFMD occurrence have been recognized. Using the Bayesian Maximum Entropy (BME) model and self-organizing map (SOM) algorithm, the number of HFMD cases has been shown to have a close relationship to monthly precipitation in Mainland China [2]. Child population density (CPD) and climatic factors were the potential determinants of HFMD incidence in most areas of the Mainland China [3]. Weekly mean temperature and cumulated rainfall are significantly associated with HFMD incidence with a time lag of 1–2 weeks in Singapore [9]. A higher risk of transmission is associated with temperatures in the range of 70°F to 80°F, higher relative humidity, lower wind speed, more precipitation, and greater population density in China [12]. In Japan, a series study found that ambient temperature and relative humidity were significantly linked with increased HFMD occurrence [17]. The association between climate variables and HFMD also has been examined in many provinces and cities of China, such as Beijing [18], Hong Kong [19], Guangdong province [20], Shenzhen [21] and Guangzhou city [22].

In previous studies, few studies have discussed the socio-economic exposures as the risk factors of HFMD occurrence at a space scale, not to mention combined with climate exposures. Methodologically, the spatial autocorrelation is prevalent in the data in social and economic sciences [23]. The neglect of the spatial autocorrelation could result in a biased and under-performing model in health risk assessment [24,25]. In spatial epidemiology, researchers are concerned more about the spatial distribution pattern of the epidemic risk more than just identifying risk factors, so it is important to predict the spatial distribution of the HFMD risk.

To address the problems and challenges mentioned above, both climate and socio-economic exposure factors were selected as potential determinants to explore the spatial risk factors of HFMD occurrence in Mainland China. We built an autologistic regression model that takes spatial autocorrelation effect of variables into account to identify the risk factors with OR values and model the spatial distribution of the HFMD risk over Mainland China at the county level. The study revealed the local variations of HFMD epidemic risk at county level by mapping the probability of disease occurrence in each geographic unit.

## Methods

### Data

**Figure 1:**
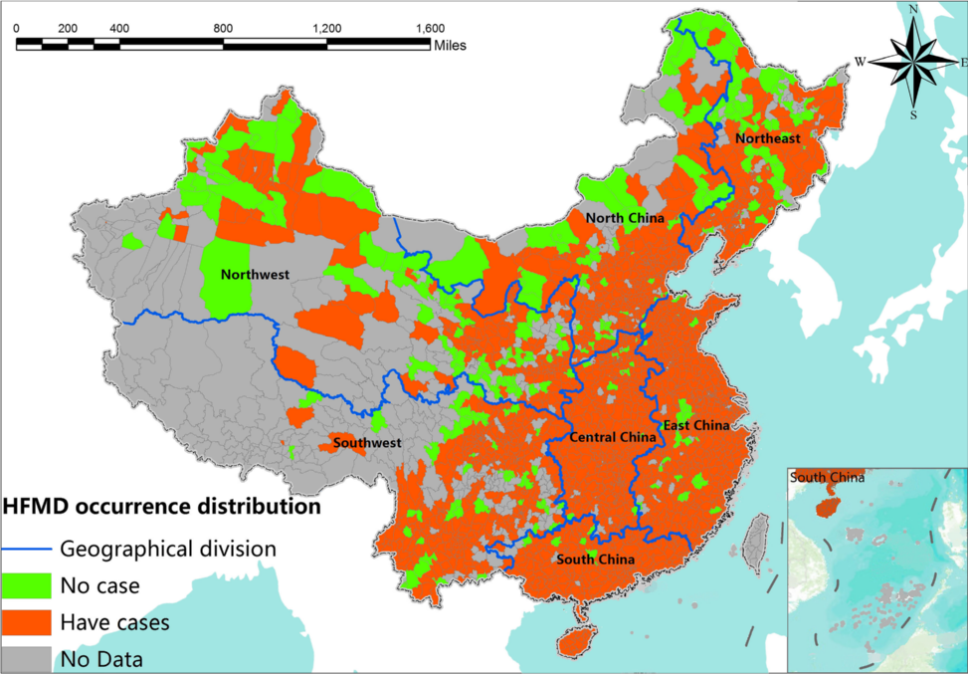
The spatial distribution of HFMD occurrence at county level in Mainland China in May 2008.

The original HFMD reported data consisted of daily number of disease cases at the county level from May 1, 2008 to March 27, 2009. The data were provided by the Chinese Center for Disease Control and Prevention (CDC). Our study focused on whether a county had any HFMD cases or not and the potential risk factors of the disease occurrence. As the dependent variable, if there are any HFMD cases in a county, it is labeled as true or 1, otherwise false or 0 [26]. The disease record in May 2008 had the most amounts of data (disease variable = 1), 87.19% of the spatial units were reported having cases. The disease record in May 2008 also had the highest number of monthly cases. Thus, the data of May 2008 was chosen as the study data in this experiment. Figure 1 shows the spatial distribution of the HFMD occurrence in Mainland China in May 2008. A total of 1,975 geographical units have valid data, where 1,722 of them were reported to have cases. The study area has been divided into seven districts, North China, East China, South China, Central China, Northeast, Southwest and Northwest.

The monthly climate data of May 2008 were provided by the China Meteorological Data Sharing Service System. The original data were collected from 727 meteorological stations over the whole area of China. We used ordinary kriging interpolation [27] and spatial aggregation technology to estimate each county’s mean value of the climate factors. Seven routine climate variables were used as candidates to explore the climate risk factors of HFMD occurrence [3]: monthly average wind speed, monthly average precipitation, monthly average temperature, monthly average temperature difference, monthly average atmospheric pressure, monthly average sunshine duration and monthly average relative humidity.

We also collected the social and economic data as potential risk factors for HFMD occurrence from the City (County) Social Economic Statistical Yearbook of China, the Regional Statistical Economic Yearbook of China and the Urban Statistical Yearbook of China in 2008. Unlike the climate data, the socio-economic factors were for the entire year of 2008. There is no monthly data available. The candidate socio-economic exposed indicators include population density, the proportion of student population, per capita household savings, the number of hospital beds per capita, industrial output value, number of industrial enterprises, product GDP per capita, number of telephones per capita and other factors.

The geographical administrative division data were obtained from the Chinese National Administration of Surveying, Mapping and Geoinformation. The data were published in 2010, so we updated, amended, merged and split the original vector data according to the latest administrative divisions’ code published by the National Bureau of Statistics of the People's Republic of China (http://www.stats.gov.cn, as of December 31, 2008), to guarantee the data were consistent with the HFMD records in every unit. As a result, HFMD data, climate data, and socio-economic data were all geo-linked to the vector geographical administrative division data in a geo-spatial database.

### Logistic and autologistic regression model

Logistic regression model has been widely used in epidemiology to explore the risk factors of disease [12,14-16,28]. The ordinary logistic model is the most common method in a case–control study. The dependent variable of the model is expressed by a binary classification variable where one indicates the true (having case), and zero indicates the false (no case). The independent variables of the model are described by a series of natural and socio-economic potential exposed factors. The model calculates the probability of the occurrence of an event, uses independent variables as the predictor values that are continuous or categorical variables. For an ordinary logistic regression, the form of the model is given by equation (1) [29,30]:

(1) $$\mathrm{In}\left(\frac{P_{i}}{1-P_{i}}\right)=\beta_{0}+\beta_{1}x_{1,i}+\cdots +\beta_{n}x_{n,i}$$

where *P*_*i*_ (probability of the occurrence of a disease) is the expected value of the dependent variable *y*_*i*_ (so that *y*_*i*_ = 1 if a sample has disease case and *y*_*i*_ =0, otherwise), *x* is the independent variables (potential exposed factors), *β* is the estimated coefficient, *i* is the index of the records, such as a geographical unit. Significance levels for variables to be included in the model are often set as 0.05 or 0.1.

The autologistic regression is the most widely used one for modeling spatially correlated presence/absence data. Indeed, many studies have demonstrated the usefulness of the autologistic regression in modeling binary data with observed covariates. The autologistic regression model is a special case of the general logistic models. It was introduced by Besag et al. (1974) [31]. The model introduces a spatial autocorrelation term in the form of weighting coefficients and solves the problem of spatial autocorrelation effects in the process of statistical analysis. We can express the conditional probability of the occurrence of a disease using equation (2) [32-35]:

(2) $$P_{i}\left(y_{i}=1|\beta_{0},\beta ,r\right)=\frac{\exp\left(\beta_{0}+\beta_{1}{x_{1}}_{,i}+\ldots +\mathrm{rAuto}\;{\mathrm{cov}}_{i}\right)}{1+\exp\left(\beta_{0}+\beta_{1}{x_{1}}_{,i}+\ldots +\mathrm{rAuto}\;{\mathrm{cov}}_{i}\right)}$$

The predicted result *P*_*i*_ denotes the probability of an event occurring for every geographic unit. *x* is independent variables. *Autocov* is the autocovariate variable. *β* and *r* are the coefficients of variables in the equation. *i* is the index of the geographical units.

Spatial autocorrelation is frequently encountered in spatial data. Typically, disease occurrences and spatial risk factors are positively autocorrelated such that nearby units in space tend to have more similar values than would be expected by random chance. Thus, models that ignore the spatial autocorrelation may be inappropriate because they might overestimate the importance of environmental variables [36]. In addition, models that ignore spatial autocorrelation effect could include variables that have little or no relevance to the response variable, creating false conclusions in modeling spatial distribution of diseases. This problem could be solved by incorporating spatial autocorrelation (autocovariate) into logistic regression models, which would result in model improvements such as increased predictive accuracy and model versatility [37].

The ordinary binary logistic model is modified to incorporate any spatial autocorrelation between geographic units by incorporating an autocovariate variable. The probability of the event occurring in one geographic unit is higher, if it is also present in the neighboring units due to the spatial autocorrelated effect. The autocovariate variable can be calculated from the predicted probabilities of occurrence, which is estimated by an ordinary logistic regression model, using the equation (3) [29,33,34]:

(3) $$\mathrm{Auto}{\mathrm{cov}}_{i}=\frac{\sum_{j=1}^{k_{i}}w_{\mathrm{ij}}{\hat{P}}_{j}}{\sum_{j=1}^{k_{i}}{w_{i}}_{j}}$$

The autocovariate variable (*Autocov*_*i*_) is a weighted average of the probabilities of the geographic units amongst a set of *k*_*i*_ neighbors of the geographic unit *i.* A method of with a certain distance (20000 m) of the centroid is used to define the neighbors of the geographic unit *i* in this study. The spatial weight between the geographic unit *i* and *j* is *w*_*ij*_*= 1/h*_*ij*_, where *h*_*ij*_ is the Euclidean distance between the centroids of geographic unit *i* and *j*, and ${\hat{p}}_{j}$ represents the probability estimated by the ordinary logistic regression model [29,33]. The autocovariate variables were incorporated into the ordinary logistic regression formula stepwisely until each parameter was statistically significant. We used the statistical software SPSS 19 to solve the ordinary logistic regression model and evaluate the model results. ArcGIS 10 was used to process the autocovariate variables data, conduct spatial analysis and create the thematic maps of risk factors and disease risk.

### Odds ratio

Odds ratio (OR) is widely used to measure the risk of a disease exposure to a determinant [38-40]. The odds ratio is the ratio of the odds that a case has been exposed to a risk factor is compared to the odds for a case that has not been exposed, using equation (4).

(4) $$\mathrm{OR}=\frac{\mathrm{odd}s_{\;\exp\mathrm{osed}}}{\mathrm{odd}s_{\mathrm{un}\;\exp\mathrm{osed}}}=\frac{p_{\;\exp\mathrm{osed}}/\left(1-p_{\;\exp\mathrm{osed}}\right)}{p_{\mathrm{un}\;\exp\mathrm{osed}}/\left(1-p_{\mathrm{un}\;\exp\mathrm{osed}}\right)}$$

where *P* is the probability of the event in a group. An OR greater than 1.0 indicates that the condition or event is more likely to occur in the first group (exposed), and an OR less than 1.0 indicates that the condition or event is less likely to occur in the first group (exposed) [40]. Logistic regression is one method to generalize the odds ratio beyond two binary variables. OR values can be obtained directly by a logistic regression model, using equation (5):

(5) $$OR_{x}=e^{\beta }$$

where *β* is the coefficient of the environmental variable *x*[41]. In epidemiology, an OR value higher than 1.0 indicates that the variable (exposure factor) is a positively correlated risk factor, lower than 1.0 means a negatively correlated risk factor and equal to 1.0 indicates an unrelated factor. For instance, it is common to describe the OR value of two in terms of a twofold risk of developing a disease compared with the reference group [40]. We obtained the OR value of every exposed factor and identified the risk factors of HFMD using this method.

### Identification of risk variables

Method for identifying the risk factors was carried out in SPSS 19.0. All the climate and socio-economic variables (Additional file 1) were included into the analysis to determine which of them were significantly associated with HFMD occurrence. Two types of determinants are suspected potentially to cause HFMD, the climatic and the socio-economic. The candidate climatic variables reflect the monthly average status of wind speed, precipitation, temperature, temperature difference, atmospheric pressure, sunshine duration, and relative humidity. Socio-economic variables were taken into account to identify the significant related variables to the HFMD occurrence, which reflect the population, regional comprehensive economy, agriculture, industry and investment, education, public health and social security of the administrative division unit. We conducted transformation processing for some socio-economic indicators before modeling to guarantee the comparability between administrative division units. For example, the number of hospital beds is divided by the population into a number of beds for every million people. All variables were standardized.

**Figure 2:**
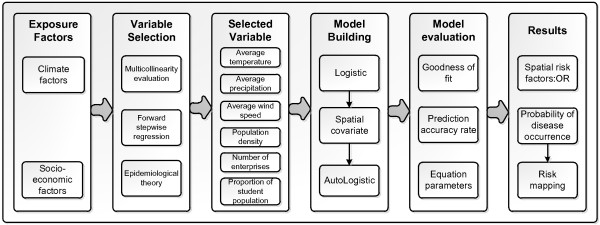
Experiment flow chart.

Figure 2 is the experiment flow chart that shows how to select exposure variables and use them to build the models. The first criterion of selection is the impact of multicollinearity. Multicollinearity is a statistical phenomenon in which two or more predictor variables in a multiple regression model are highly correlated [42-44]. Multicollinearity can lead to the meaning of the equation parameters unreasonable, cause significant test variables lose significance and exclude important explanatory variables from of the model. To decrease such influence, we calculated the variance inflation factor (VIF) and tolerance for each climate variable and socio-economic variable to assess the multicollinearity. In general, the smaller the tolerance and the larger the VIF is, the more serious multicollinearity is [45]. In ordinary logistic models, it is usually taken 10 as the threshold value of VIF. With consideration that the study area is too large and a lot of spatial factors need to be identified, we use a VIF less than 15 as the threshold to choose variables without multicollinearity effect in our case.

Secondly, we used the stepwise regression method to exclude the variables without statistical significance to establish the best predictive regression model. In statistics, stepwise regression includes regression models in which the choice of predictive variables is conducted by an automatic procedure. Here, we used forward stepwise regression. At the beginning, the model is without any independent variable. Then, introducing variables into the model one by one, each time selecting one variable with the smallest p value (significance) and that the p value < *α*_1_ (threshold value) into the model. Once we introduced a variable into the regression model, we needed to investigate whether the model had a variable with p value > *α*_2_ (threshold value). If it existed, then it was excluded from the model, and the model was re-fitted. Introducing the variables step by step until there were no more variables can satisfy the above conditions. In this experiment, we set *α*_1_ =0.05 and *α*_2_ =0.1 as threshold values when using ordinary logistic regression. As a result, the selected variables were statistically significant.

## Results

### Spatial distribution of the risk factors

In May 2008, among the recorded geographical units, 12.06% were reported to have no HFMD cases (dependent variable =0), and 87.94% were reported to have more than one HFMD case (dependent variable =1). Figure 1 displays the spatial distribution of the HFMD-dependent values in Mainland China. The distribution of HFMD occurrence shows a strong spatial aggregation that mainly concentrates in eastern, southern and central China. Other regions near the center of the aggregation also present a serious aggregation, such as the south-eastern part in north China and the southern part in northeast China. It is easier to have HFMD cases when the surrounding counties have cases. This spatial autocorrelation characteristic makes it necessary to consider the spatial effect in a logistic regression model [24].

**Figure 3:**
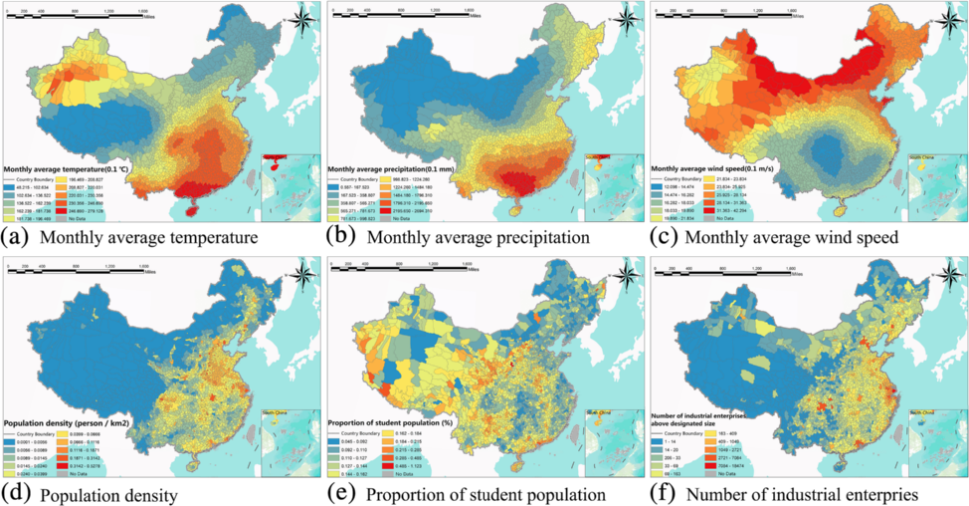
**The spatial distribution of selected exposure factors in the regression model in China in May 2008. (a)** Monthly average temperature. **(b)** Monthly average precipitation. **(c)** Monthly average wind speed. **(d)** Population density. **(e)** Proportion of student population. **(f)** Number of industrial enterprises above designated size.

The significant risk factors identified by the flowchart in Figure 2 are monthly average temperature, monthly average precipitation, monthly average wind speed, population density, number of industrial enterprises above designated size and the proportion of student population. Figure 3 shows the spatial distribution of the identified HFMD risk factors. No data regions refer to Taiwan, Hong Kong and the islands in the south, which are not in our study area of Mainland China. The spatial variations of climate factors are large and have strong spatial heterogeneity among the whole region. For example, the difference between the maximum and minimum average temperature is 23.09°C and the standard deviation is 42.19°C. The same situation can be observed for the average precipitation and the average wind speed, which led to significant spatial heterogeneity. High temperature and precipitation are mainly concentrated in eastern, northern and southern China, whereas the values tend to be lower in western and north-eastern China in Figure 3a and b. Socio-economic factors involving population density and the number of industrial enterprises above designated size show a similar trend across the area, decreasing from south-eastern to northwestern China (Figure 3d and f). The spatial trend of the average wind speed and the proportion of the student population are different from the previous. The average wind speed decreased from the north to the south, and the proportion of students increased from the northeast to the southwest (Figure 3c and e).

**Figure 4:**
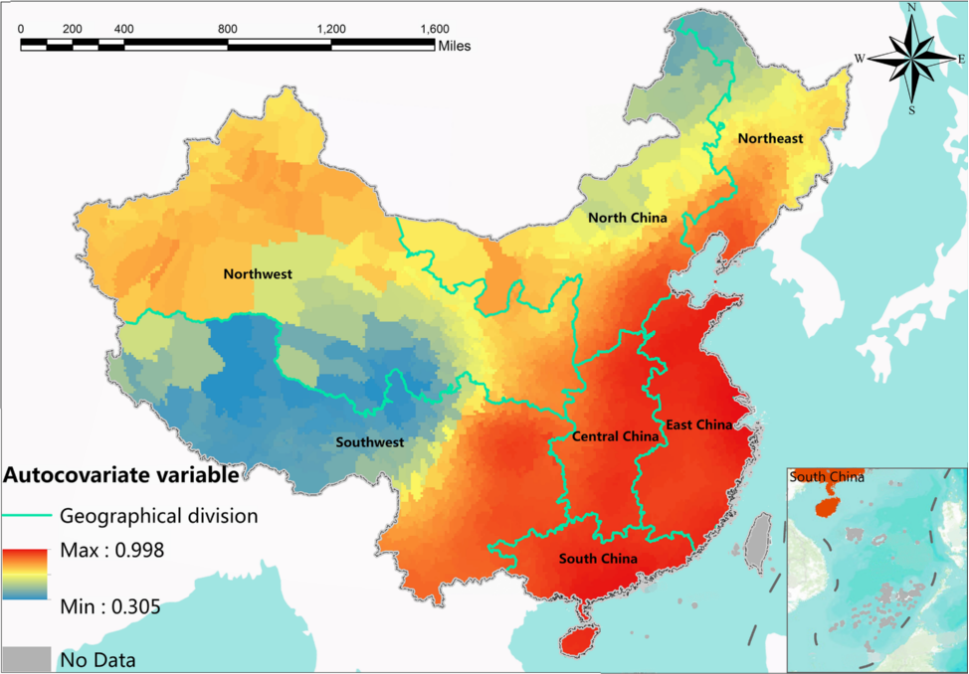
The spatial distribution of the autocorrelation term: the autocovariate variable.

Figure 4 shows the spatial distribution of the autocovariate variable in equation (3), which represents the residual spatial autocorrelation term in the autologistic regression model. “No data” regions refer to Taiwan, Hong Kong and the islands in the south. Introducing the spatial autocovariate variable reflects the first law of geography expressed as spatial autocorrelation [46]. It is actually a process of data smoothing, reducing local spatial differences between geographical units to present the inherent spatial difference and tendency. The autocovariate variable has the same unit of the dependent variable, which also represents the probability of the disease occurrence, but it is just a macro spatial trend. Figure 4 shows that the spatial distribution of probability of HFMD occurrence has a strong spatial tendency and heterogeneity, which presents a transitional and gradual change throughout Mainland China. The probability of disease occurrence gradually increases from the northwest to the southeast. The probability is very high in central, southern and eastern china, which is consistent with what we observed in Figure 1 that these areas were hit hardest by the HFMD outbreak.

### Validation of the models

In our case, the model was evaluated based on the goodness of fit, the prediction accuracy, and the equation coefficients of model [33,35,47]. An autologistic regression model needs the autocovariate variable first, and this step requires the prediction results of the ordinary logistic regression model. The ordinary logistic regression model is an intermediate step in building a spatial model (Figure 2). The performance of the logistic and autologistic regressions models are listed to indicate that they both pass the significance tests. In addition, we selected 30% of samples from all geographical units as the verified dataset using stratified random sampling [48,49], and the remaining 70% of samples (1383 records) were used for model building. For the binary value of disease, we stratified the whole area into having case and no case stratas, and selected the samples based on the simple random sampling method in the two stratas respectively.

### Goodness of fit

Table 1 summarizes the overall model statistics of the logistic and autologistic regression models. A key starting point could be the model chi-square whose value provides the usual significance test for a logistic regression. It is a difference between the best-fitting model and the null hypothesis in which all the coefficients are set to 0. The chi-squares values of the models measure the improvement of fitness due to the inclusion of independent variables into the regression. A high value indicates that the occurrence of disease is far less likely under the null hypothesis (without any influencing parameters) than the full regression model where the parameters are included [47]. Both models are highly significant with parameters. The goodness of fit of the models was further measured by the Cox & Snell R Square and Nagelkerke R Square statistics. These are often called pseudo-coefficients of determination. The higher the statistic values are, the better the model fits the observations. Both of the models pass the significance test. The Relative Operating Characteristic (ROC) is used to compare a Boolean map of “reality” (the presence or absence of disease) with the probability map. The ROC value ranges from 0.5 to 1, where 1 indicates a perfect fit and 0.5 represents a random fit. ROC is the reporting area under the Receiver Operating Characteristic Curve which we obtained in SPSS. In our case, the ROC of the logistic model is 0.816, and the ROC of the autologistic model is 0.817, which indicate a high correlation between the independent and dependent variables. In summary, both of the autologistic regression model and the ordinary logistic regression model have a good goodness of fit.

**Table 1:** Summary statistics of the logistic and autologistic regression model

**Statistics**	**Logistic**	**Autologistic**
Modeling sample proportion	70%	70%
Number	1383	1383
Model chi-square	206.806	209.640
Cox & Snell R Square	0.139	0.141
Nagelkerke R Square	0.266	0.270
ROC	0.816	0.817

### Prediction accuracy

Prediction accuracy represents the accuracy of the predicted results of the dependent variable. The larger the value is, the better the prediction is. It is obtained from the contingency table between the recorded data and the predicted result. Table 2 summarizes the prediction accuracy of the spatial autologistic regression model, including the no-case region (no HFMD case), the having-cases region (having HFMD case) and the whole region (all samples). For the prediction results of verification data, the accuracies of the no-case region, the having-cases region and the whole region are 63.95%, 80.24% and 77.87% respectively.

**Table 2:** The prediction accuracy of the spatial autologistic regression model

	**Accuracy (%)**	
Modeling (70% samples)	No-case region	67.66
	Having-cases region	79.93
	The whole region	78.45
Verification (30% samples)	No-case region	63.95
	Having-cases region	80.24
	The whole region	77.87

### Equation coefficients

The parameters of the spatial autologistic model are given in Table 3. The selected six independent variables are all statistically significant (sig. in logistic) in the forward stepwise logistic regression models. All the values of significance level are less than 0.05 in the ordinary logistic model except for average wind speed, for which the significance of average wind speed is less than 0.1. When building the autologistic regression model based on these selected variables, we used forced regression other than forward stepwise regression because adding new spatial autocorrelation variables might lead to the significance of variables increase and exclude related variables we have selected. As a result, we obtained the regression coefficients of every variable and their OR values, as well as the OR values of the spatial autologistic regression model between the 95% confidence interval. The result shows that the OR values of precipitation, temperature, wind speed, population density, proportion of the student population and number of industrial enterprises above designated size are all greater than 1, which indicates that these variables are significant risk factors that are positively related to the occurrence of HFMD. The OR value of the added spatial covariate is the greatest among all variables, which indicates that it is a positively correlated risk factor to the occurrence of HFMD.

**Table 3:** The OR values obtained for the independent parameters

**Independent parameter**	**Sig. in logistic**	**Coefficient in autologistic**	**OR**	**95% CI of OR**
Average temperature	0.000	0.321	1.379	0.863-2.205
Average precipitation	0.002	0.361	1.434	1.044-1.969
Average wind speed	0.074	0.152	1.186	0.881-1.598
Population density	0.001	0.670	1.953	1.215-3.140
Number of above-scale enterprises	0.003	2.873	17.699	1.969-159.095
Proportion of the student population	0.020	0.252	1.286	1.209-1.607
Constant	0.000	0.223	1.250	——
Autocovariate variable	——	2.934	18.800	0.614-575.604

In addition, the added spatial variable (autocovariate variable) has a significant reduction of the contribution of the constant in the equation (from OR = 17.25 to OR = 1.25). This reduction indicated that the added spatial variable expresses the spatial effect in the constant term and reduces the residual error of the constant. Therefore, it is very meaningful to introduce the residual spatial autocorrelation into the model because the spatial autologistic regression model excavates inherent errors that are caused by spatial autocorrelation effect in the ordinary logistic regression model.

### Spatial distribution of the probability of HFMD occurrence

**Figure 5:**
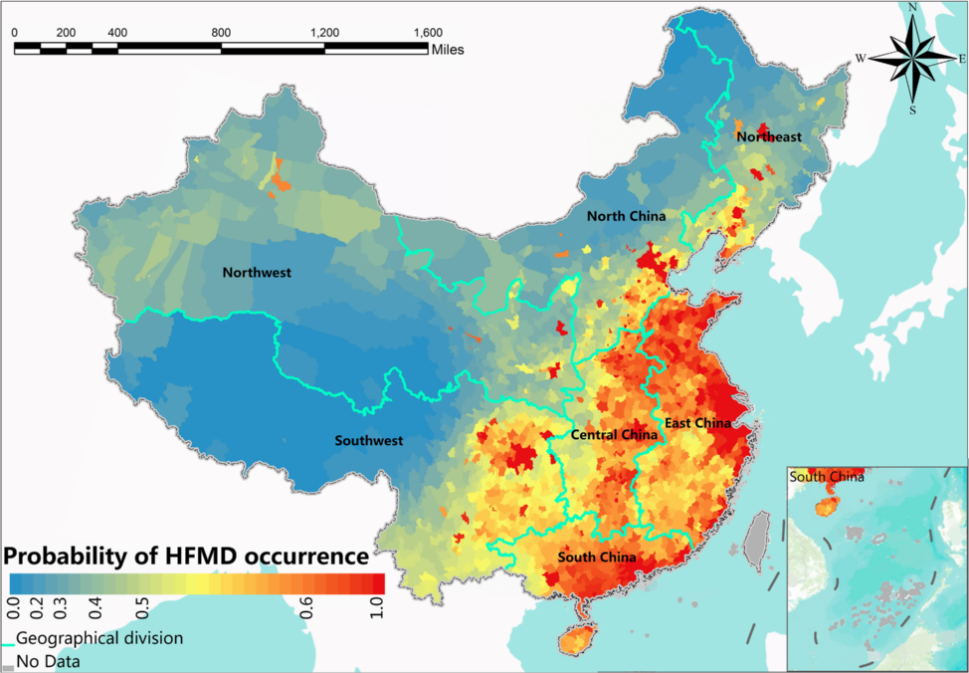
The spatial distribution of the probability of disease occurrence in Mainland China.

The OR values of potential determinants were used to identify spatial risk factors. We estimated the *P*_*i*_ in equation (2) at each county. Figure 5 shows the probability *P*_*i*_ of disease occurrence of every county in Mainland China to represent the local epidemic risk level. No data regions refer to Taiwan, Hong Kong and the islands in the south. In local space scale, *P = 0.5* is taken as a reference standard based on the segmentation criteria in the autologistic model for whether there is disease happen or not in this region. If the *P* value is greater than 0.5, this indicates that this area is risky for HFMD under the combined effects of the risk factors. The larger the *P* value is, the more dangerous the county is. Conversely, a *P* value less than 0.5 indicates that the county is safe for HFMD. The smaller the *P* value is, the less dangerous the county is. The risk areas of HFMD in Mainland China are mainly locate in eastern, southern and central China, especially in Beijing, Tianjin, Shandong, Henan, Guangdong and Sichuan province. The spatial distribution of probability *P*_*i*_ represents more local details of the HFMD risk variations than the spatial distribution of HFMD original records does. For instance, from Figure 1, one can only find that the eastern China was hit hardest by HFMD, but one can find more local variations of the HFMD risk in the eastern China in Figure 5. Such local variations of the HFMD risk can also be found in the north, central, south, northeast, northwest and southwest regions of China. The local patterns of disease are important because the regions with high risk are worth of more attentions in the disease prevention and control.

**Figure 6:**
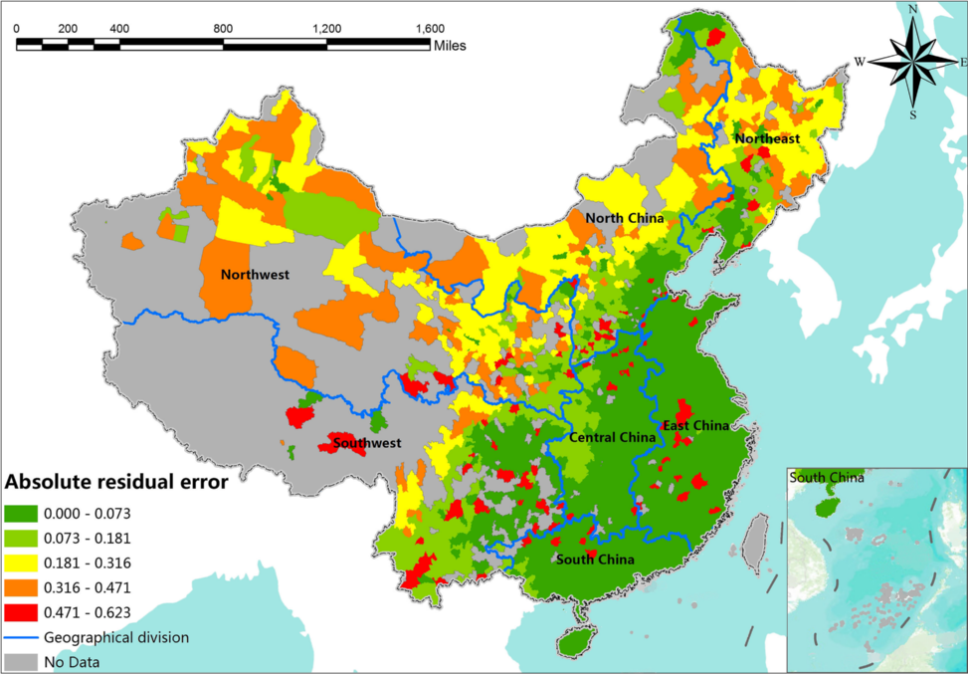
The spatial distribution of the uncertainty of the predictions in the study area.

We further obtained the absolute residual error of the estimated *P*_*i*_ where original disease records were available to evaluate the uncertainties of the risk predictions. The absolute residual error is calculated by |*P*_*real*_*-P*_*predicted*_|, in which *P* is the probability of given geographical unit. We created the absolute residual error map to present the spatial uncertainty (Figure 6). The absolute residual error map shows that the probabilities at most of the counties are well predicted with residual errors less than 0.2, especially in East, South and Central China, where there have been the most serious HFMD risk. The regions with relative high residual error are located in areas with low-risk, such as North and Northwest China.

## Discussion

### Autologistic regression model

There are often marked spatial autocorrelations in prediction residual terms in the regression analysis for public health risk assessment due to the spatial effects that are not captured in the model [46,47]. The ordinary logistic regression model is a non-spatial model, and it is based on the assumption that the relationship between disease risk and potential explanatory factors is a stationary spatial process. It is reasonable to assume that the explanatory factors and the relationship between HFMD and the potential risk factors would not change significantly across the whole region for a small and homogenous region of interest. However, the topography, climate and socio-economic factors change greatly over regions in regard to a large region such as China with a territory over 9.6 million square kilometers [50]. It is impossible to maintain the spatial stationary assumption in such a large and heterogeneous area. As a result, the neglect of the spatial effect in a regression analysis could result in a biased and under-performing model in health risk assessment [25].

The autologistic regression model is a spatial model that considers the spatial residual autocorrelation effect in the model. It introduces a spatial residual autocorrelation variable based on a rational assumption that the relationships between the risks of the exposure factors and the risk of HFMD occurrence are more similar in nearby regions. Only nearby counties are included in the spatial autocovariate variable, and every included county is given a weight according to its spatial distance to the destination county. The spatial autocovariate variable also reflects the global spatial trend of the probability the HFMD occurrence, as is shown in Figure 4.

The autologistic regression model also reduces the contribution of the residual significantly, which reflects the spatial inherent residuals, by introducing a spatial autocorrelation variable. The constant in the regression model is the prediction residual error of the model. The smaller the contribution of the constant is, the better the explanatory power of the model is. By introducing the spatial autocovariate variable, the contribution of the constant is reduced significantly in the autologistic model. It changes from OR = 17.25 (for logistic regression) to OR = 1.25 (for autologistic regression). However, the spatial autocovariate variable can be comprehended as the spatial inherent residual to reflect spatial effect in space data, which can reduce bias in health risk assessment. The spatial autocovariate variable helped to remove inherent residual errors from the ordinary logistic regression model.

The main shortcoming of the autologistic regression model is that it’s only suitable for the binary or multiple category dependent variables. The other one is that the autologistic regression model does not have a spatio-temple type so far, which cannot satisfy the need for the analysis of spatio-temporal variation of disease risk.

### Identification of risk factors

This study used an autologistic regression model to identify risk factors of HFMD in Mainland China from 29 potential exposed variables (Additional file 1). The results indicate that both climate and socio-economic factors were significant spatial risk factors for HFMD occurrence in Mainland China in May 2008. The significant spatial risk factors are monthly average temperature (OR = 1.379), monthly average precipitation (OR = 1.4354), monthly average wind speed (OR = 1.186), population density (OR = 1.953), the number of industrial enterprises above designated size (OR = 17.699) and the proportion of student population (OR = 1.286). All the risk factors are positively correlated to the occurrence of HFMD.

Our study revealed that the climate factors, such as the air temperature, relative humidity, wind speed and the precipitation, are risk factors associated with the occurrence of HFMD, which is consistent with the conclusions of previous studies in Mainland China, Taiwan and Hong Kong [2,4,19].

To our knowledge, this is the first study to explore the socio-economic factors of HFMD from a spatial analysis point of view. It is worth mentioning that introducing spatial socio-economic factors into HFMD risk assessment can explain the spatial pattern of HFMD risk more comprehensively and objectively, than previous studies that are mainly concerned with climate risk factors. We found that in addition to the climate factors, population density, the number of industrial enterprises above designated size and the proportion of student population had significant contributions to the risk of HFMD incidence. The number of industrial enterprises has the largest value of OR (OR = 17.699) among all socio-economic factors, which indicates the level of industry of a county is very risky. It has been confirmed that air pollution is closely related to the industrial level, which would lead to many epidemic diseases and weaken the immunity especially between children and elderly people [51]. We can also find that heavy industrial areas in Figure 3f are significantly consistent with the distribution of cluster disease areas in Figure 1. It makes the value of OR much larger. This indicates that level of industrialization of a county can influence the air pollution level, concentration of PM2.5 and Children's immunity, which thereby affecting the HFMD outbreak.

The comprehensive effect of the climate and social-economic factors makes the regression model have more convincing power and application value. In addition, the autocovariate variable (OR = 18.800) also occupies an important contribution in the autologistic model. It indicates that except for the climate and social-economic factors, the HFMD occurrence risk is related to the spatial location. Thus, the autocovariate variable can be seen as the third type of risk factor addition to the climate and social-economic factors.

### The risk in local areas

In spatial epidemiology, researchers are concerned more about the degree of epidemic risk in each geographic unit (counties and cities) at the local spatial scale than the identification of risk factors. We identified the spatial risk factors of HFMD throughout the Mainland China. Another purpose of this research was to determine the combined and interactive effects of these spatial risk factors on HFMD occurrence, which can be called local disease risk. We modeled the probability *P*_*i*_ of the HFMD occurrence to each geographical unit to express the spatial distribution pattern of risk (Figure 5).

Using the probability *P*_*i*_ of the HFMD occurrence to identify the risk in local areas has many advantages over traditional epidemiological studies. First of all, compared with the original data, the high value region in Figure 5 is consistent with the distribution of cluster disease area in Figure 1. The risk areas are mainly distributed in eastern, southern and central China. Furthermore, the spatial distribution map for probability fills the missing data places where there are no HFMD collection agencies and reflects the risks in those no-data regions. Secondly, spatialized probability obviously displays more local details of epidemic risk. For instance, previously we only knew it was hit the hardest by the HFMD in the eastern China from Figure 4. Now, we can identify that the risk in the east areas is more serious, and the risk in the north is more serious than the risk in the south. There are also three stepped transitions of HFMD risk level in the southwest of China from west to east. The particular risk areas are in Beijing, Tianjin, Shandong, Henan Guangzhou and Sichuan province. Finally, identifying the local areas epidemic risk can provide valuable information regarding the allocation of public health resources for prevention and treatment purposes compared with traditional epidemiology. In this context, we calculated the probability of HFMD occurrence in each geographical unit, connected them to the spatial features and produced a disease risk hierarchical thematic map to show the spatial distribution pattern of HFMD local risk with GIS technology.

### Limitations

There are some biases in the study that may affect the results. First and most importantly, the underreporting of HFMD cases in clinics and hospitals is a potential limitation of our study because of the individual disease severity and the gap between the levels of regional medical resources [3], and regional differences in the reporting of HFMD cases can influence the dependent variable and verification accuracy. Secondly, because the socio-economic factors are annual average data, the spatial heterogeneity might be hidden or smooth, which could result in uncertainty. Thirdly, bias could come from the sampling error when choosing modeling data and validation data, which cannot be avoided. That is, if there is sampling, bias will occur. Finally, confounding bias is a widespread uncertainty in epidemiological analysis, especially in spatial epidemiology in which socio-economic factors are strong predictors of the vast majority of health outcomes but when exposed to many environmental-related factors, the confounding phenomenon will be more obvious.

## Conclusions

Using a spatial autologistic regression model, we found that HFMD occurrence is heterogeneously related to the climate and socio-economic factors distributed at the county level unit over the Mainland China. The combined effect of socio-economic and climate indicators can explain the determents of HFMD outbreak more comprehensively and objectively. The spatial autologistic regression model that considers spatial autocorrelation and heterogeneity has a relative good goodness of fit and prediction accuracy of HFMD occurrence. The spatial autologistic regression model also reduces the residuals in the ordinary logistic regression model significantly. The spatial autologistic model can be used to geographically differentiate the local risk of disease occurrence by the variation of explanatory spatial risk factors. Through the spatial probability risk map, people can identify risk characteristics in local areas to determine the spatial distribution pattern of HFMD occurrence. Such conclusions could guide local public health institutes to rationally allocate public health resources and improve their preparedness for an outbreak according to region-specific conditions. As a result, the spatial autologistic regression model is effectively used to identify spatial risk factors and spatial patterns of the risk of HFMD. The experimental process and results may provide a theoretical basis, epidemic prediction and determination of the focus areas for national HFMD prevention and control in China.

## Competing interests

The authors declare that they have no competing interests.

## Authors’ contributions

YCB made substantial contributions to the conception and the design of this research, and revised it critically for intellectual content. CS did all spatial data analysis and wrote the whole manuscript. JFW suggested the method to be used, revised the manuscript. XWL participated in GIS analysis and the revision of this manuscript. All authors read and approved the final manuscript.

## Pre-publication history

The pre-publication history for this paper can be accessed here:

http://www.biomedcentral.com/1471-2458/14/358/prepub

## Supplementary Material

Additional file 1 **The spatial distribution of the potential exposed variables.** The Additional file 1 is standard Doc format. It introduces the spatial distribution of 29 potential exposed variables in this study, as reviewer suggested. Click here for file
