# Supplementary material for: Using an autologistic regression model to identify spatial risk factors and spatial risk patterns of hand, foot and mouth disease (HFMD) in Mainland China
Source: BMC Public Health. 2014 Apr 14;14:358. doi: 10.1186/1471-2458-14-358 (PMC4022446; doi:10.1186/1471-2458-14-358)

# The spatial distribution of the potential exposed variables in this study

## Overview

This study used an autologistic regression model to identify risk factors of HFMD in mainland China from 29 potential exposed variables (Table 1). The monthly climate data of May 2008 were provided by the China Meteorological Data Sharing Service System. The socio-economic factors were from the City (County) Social Economic Statistical Yearbook of China, the Regional Statistical Economic Yearbook of China and the Urban Statistical Yearbook of China in 2008. Unlike the climate data, the socio-economic factors were for the entire year of 2008. There is no change in every month for socio-economic factors. The default standardization method we used in SPSS is z-score standardization. All the variables were standardized to be dimensionless.

Table 1. The list of 29 potential exposed variables

| index | climate factors | |  | socio-economic factors |  |  |
| --- | --- | --- | --- | --- | --- | --- |
| 1 | Monthly average wind speed | | | Local telephone users end of the year | | |
| 2 | Monthly average precipitation | | | The per capita savings deposits of urban and rural residents (million) | | |
| 3 | Monthly average temperature | | | The number of hospital beds  per capita | | |
| 4 | Monthly average temperature difference | | | The number of employees end of the year | | |
| 5 | Monthly average atmospheric pressure | | | Local general budget revenue (million) | | |
| 6 | Monthly average sunshine hours | | | Local Government Budgetary Expenditure (million) | | |
| 7 | Monthly average relative humidity | | | Savings deposits of urban and rural residents (million) | | |
| 8 |  |  |  | The loan balance of the financial institutions  end of the year (million) | | |
| 9 |  |  |  | The above-scale total industrial output value (million) | | |
| 10 |  |  |  | Social fixed asset investment (million) | | |
| 11 |  |  |  | General secondary school students (person) | | |
| 12 |  |  |  | Primary school students (person) | | |
| 13 |  |  |  | Regional GDP (million) | |  |
| 14 |  |  |  | The per capita GDP (Yuan / person) | | |
| 15 |  |  |  | The first industry output (million) | |  |
| 16 |  |  |  | The second industry output (million) | | |
| 17 |  |  |  | Tertiary industry output value (million) | | |
| 18 |  |  |  | Staff and Workers in Urban Units (person) | | |
| 19 |  |  |  | Average wage of urban Staff and Workers (Yuan) | | |
| 20 |  |  |  | The population density | |  |
| 21 |  |  |  | The number of industrial enterprises above designated size | | |
| 22 |  |  |  | The proportion of student population | | |

## Maps of the variables

### 2.1 Climate variables

#### 2.1.1 Monthly average wind speed


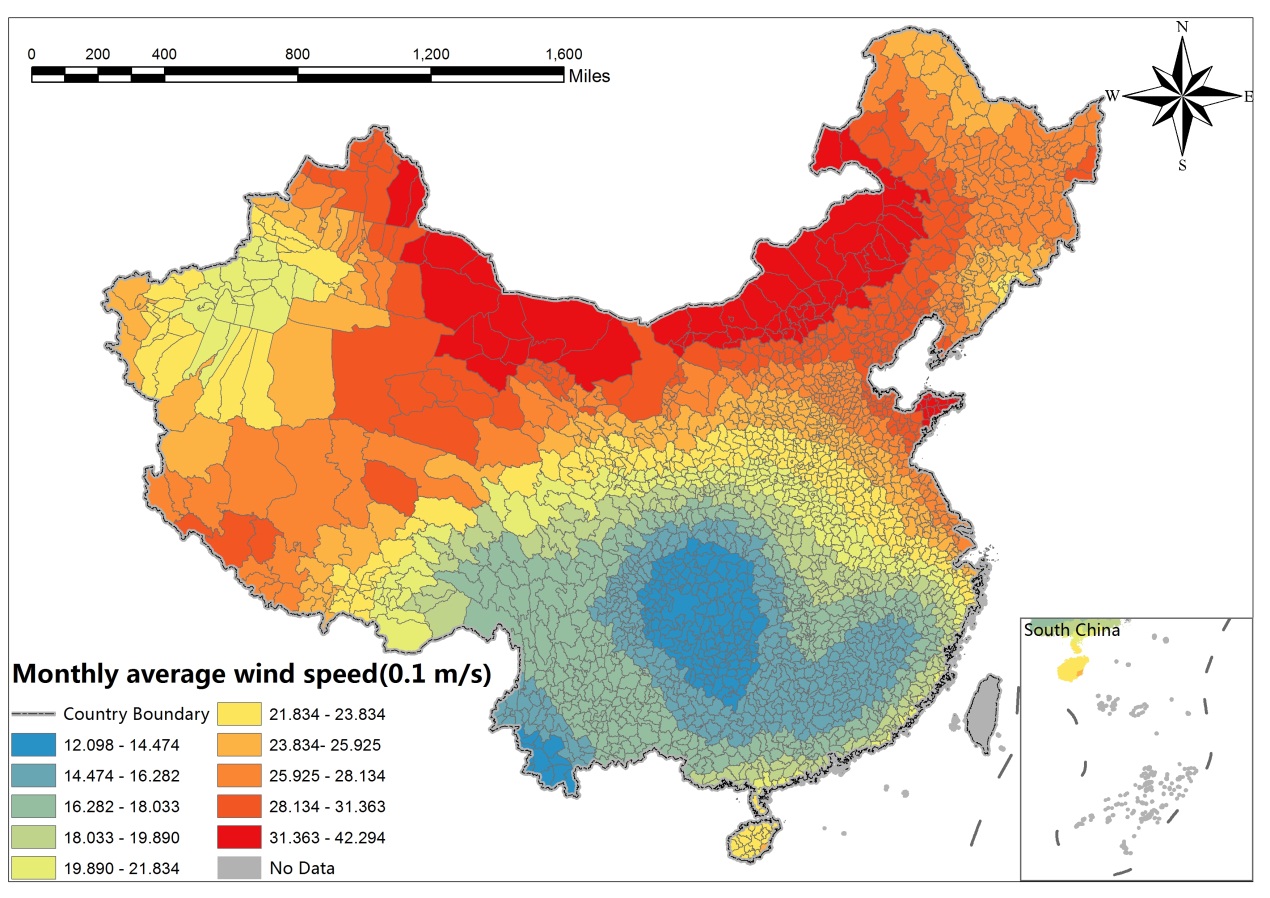


#### 2.1.2 Monthly average precipitation


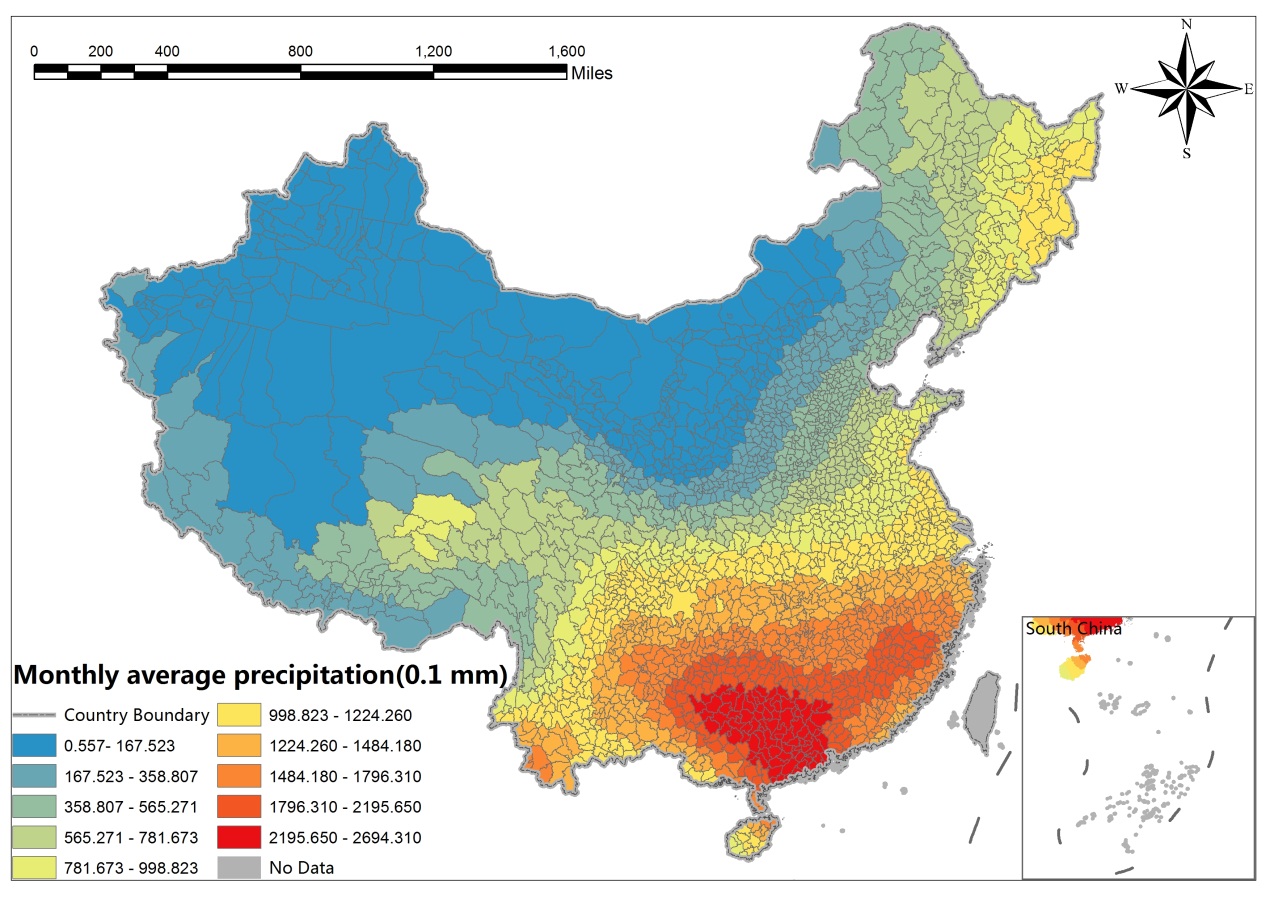


#### 2.1.3 Monthly average temperature


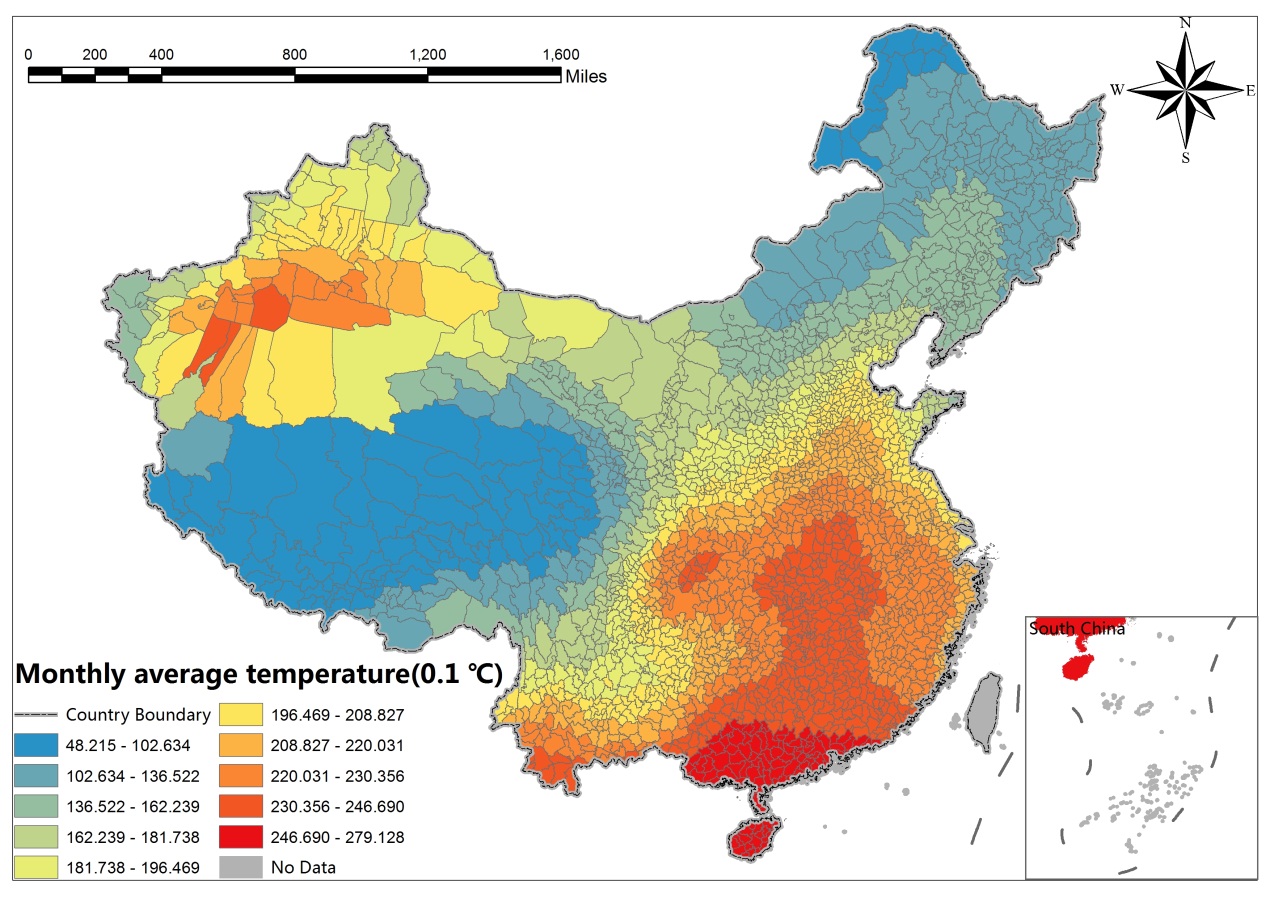


#### Monthly average temperature difference


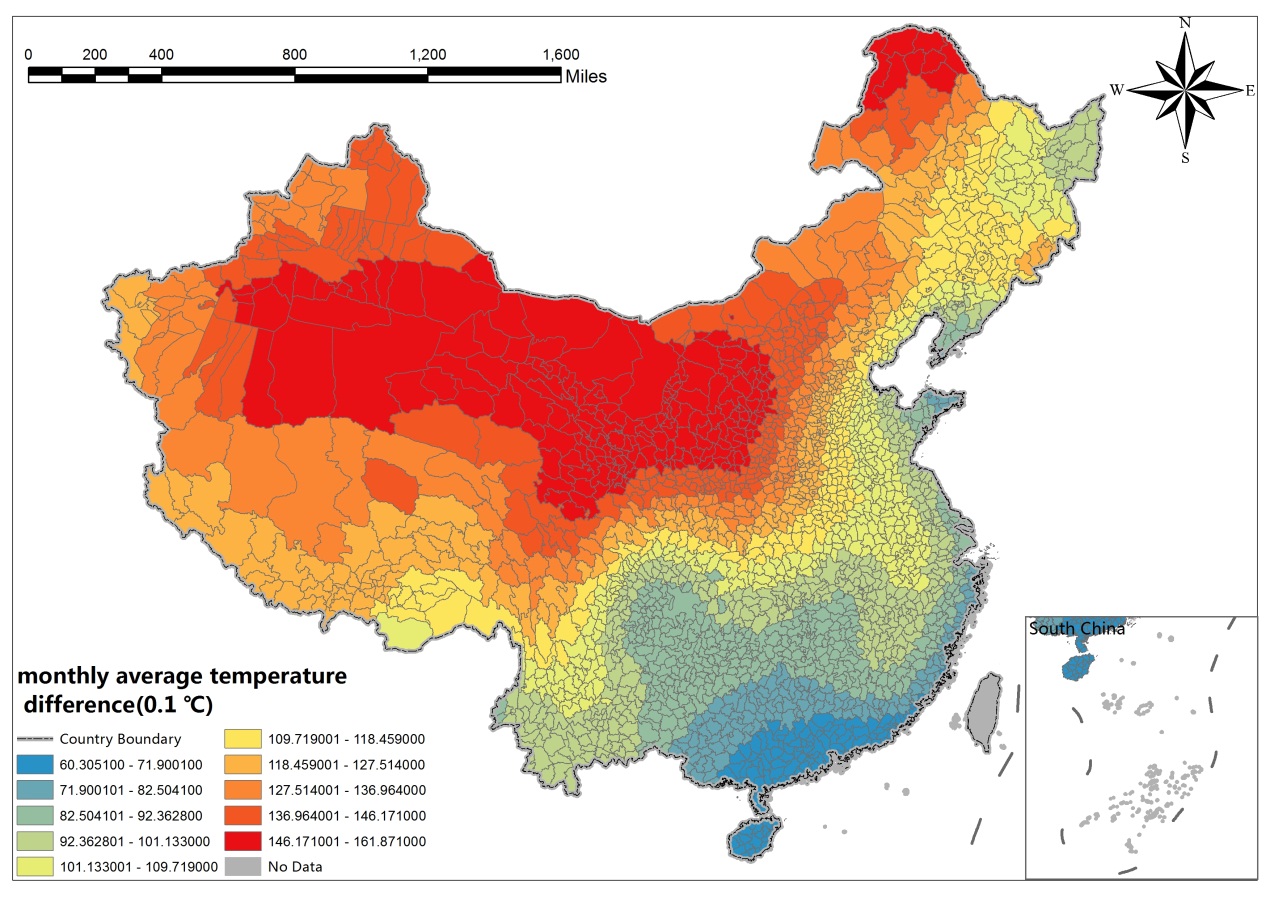


#### 2.1.5 Monthly average atmospheric pressure


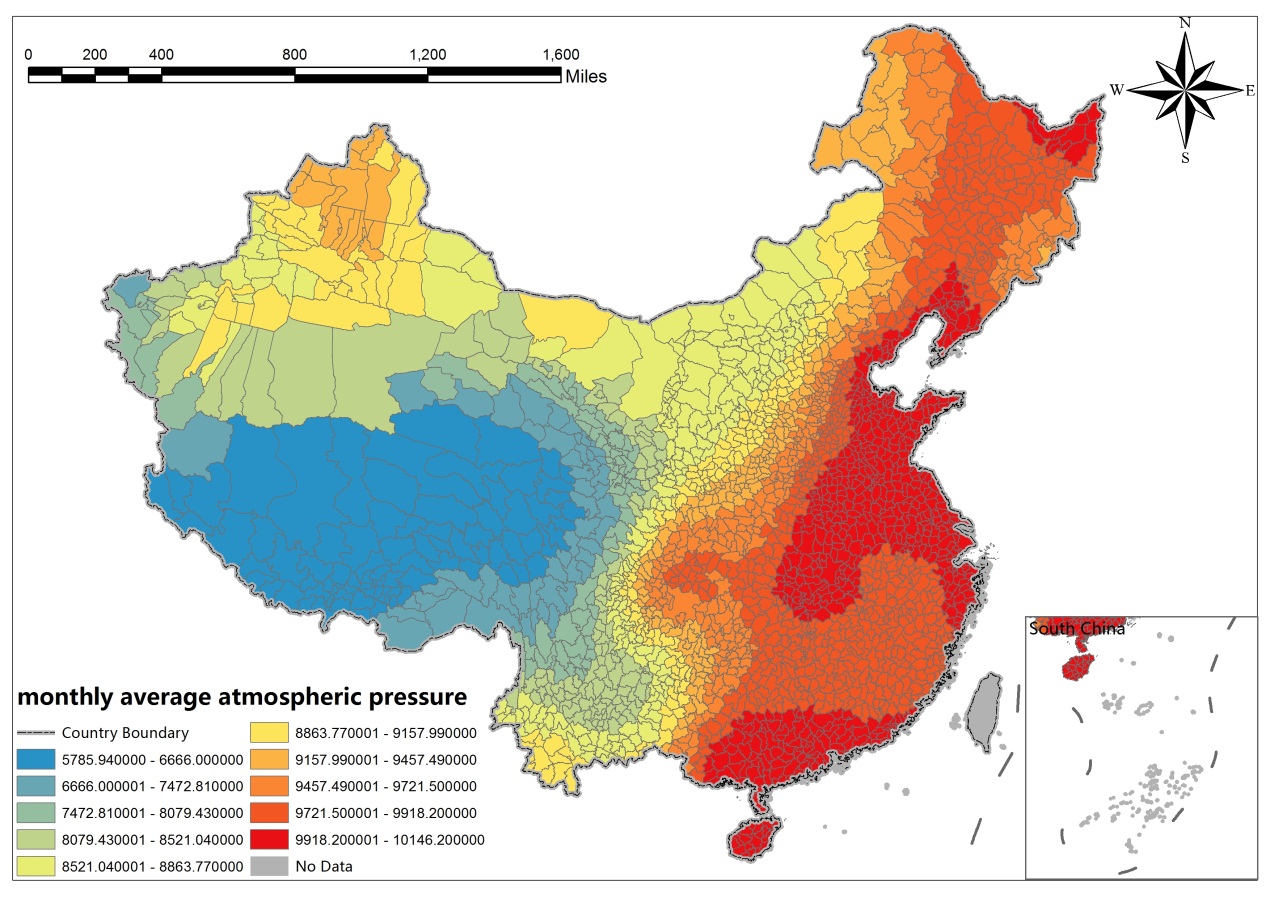


#### 2.1.6 Monthly average sunshine hours


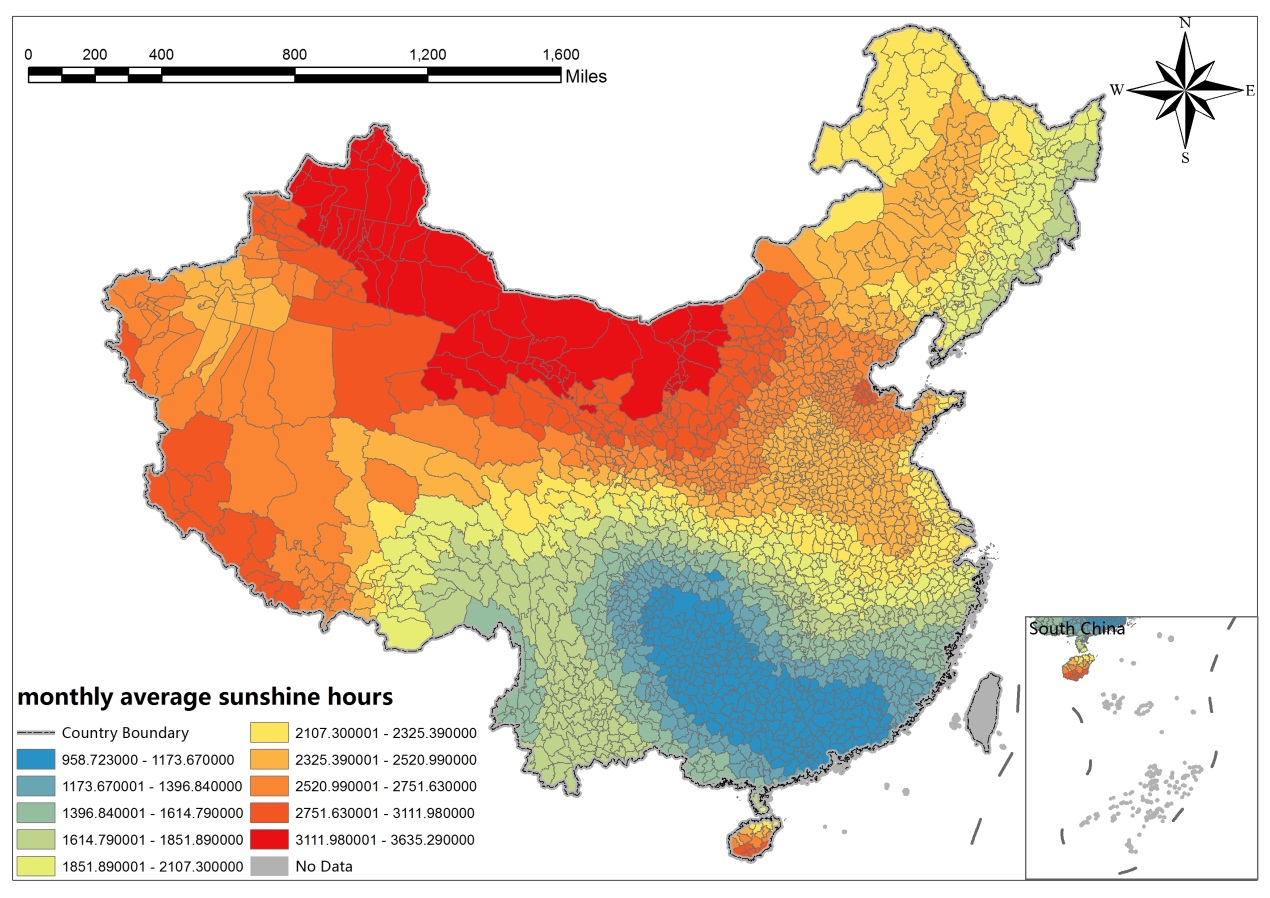


#### 2.1.7 Monthly average relative humidity


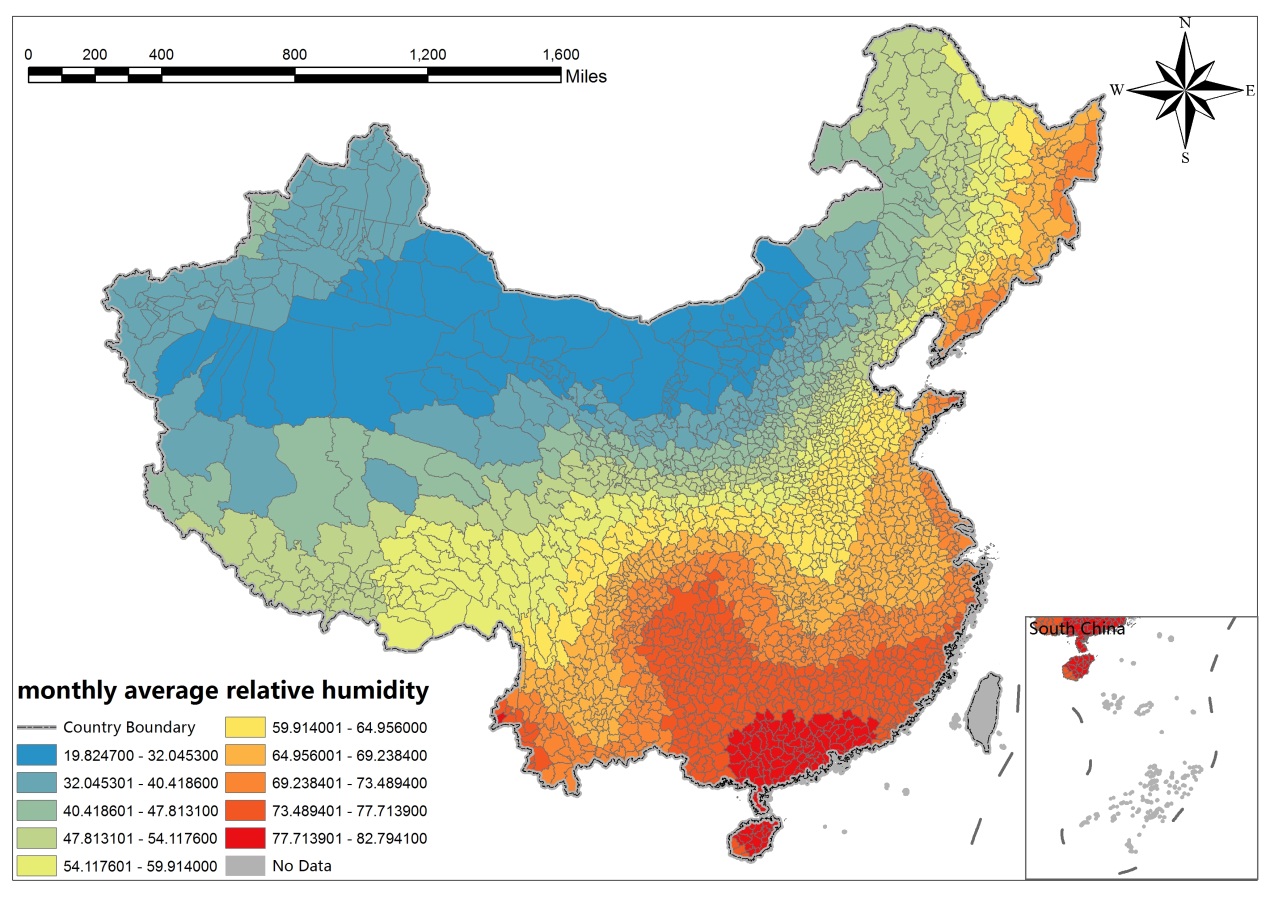


### 2.2 Socio-economic factors

#### 2.2.1 Local telephone users end of the year


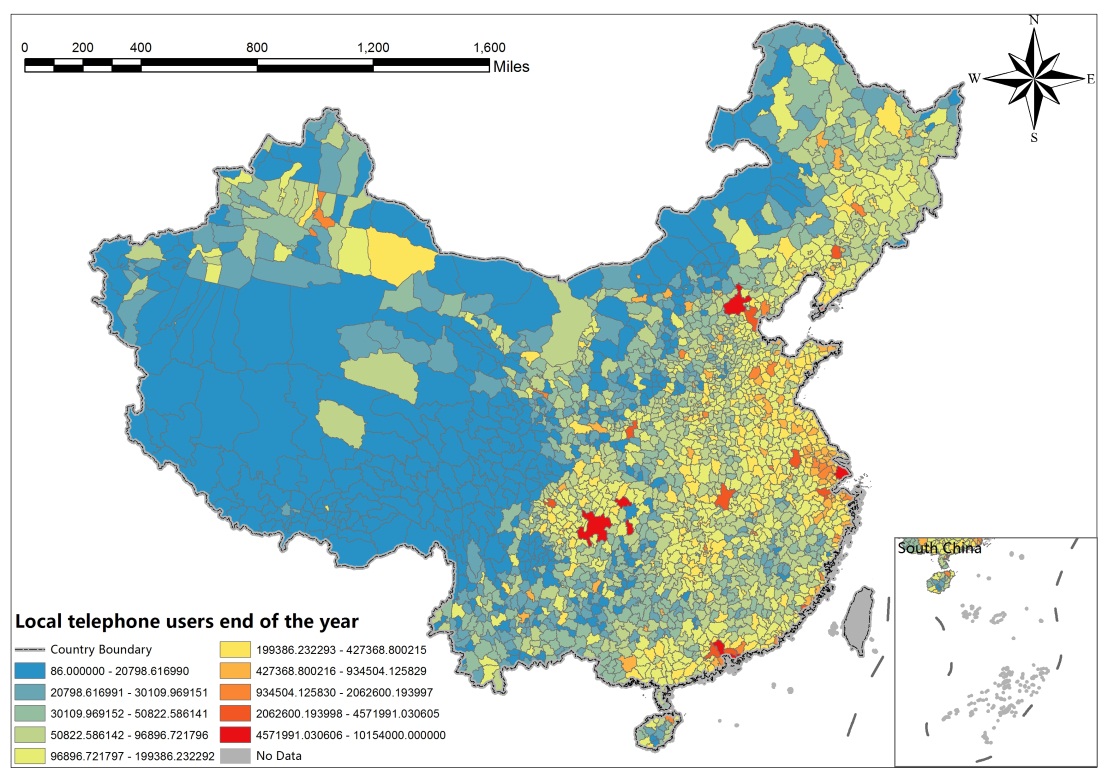


#### 2.2.2 The per capita savings deposits of urban and rural residents (million)


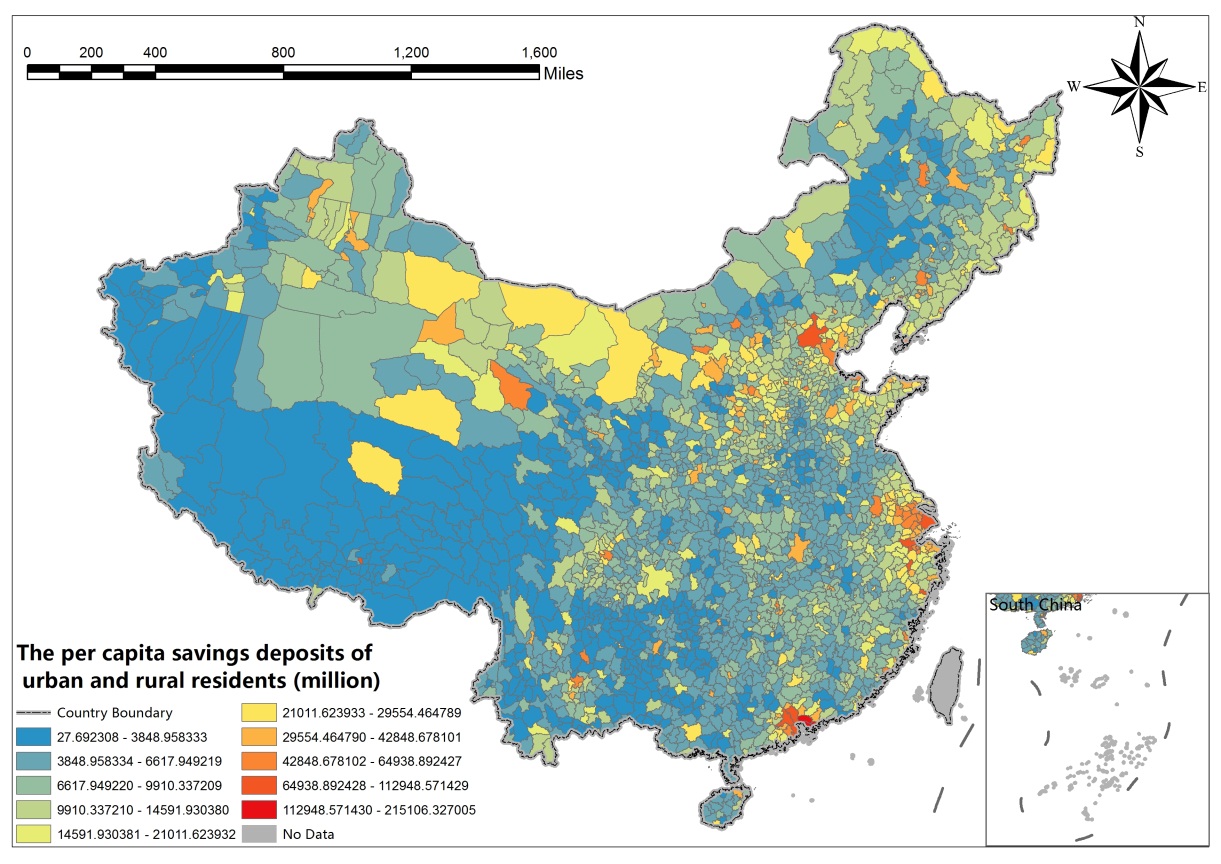


#### 2.2.3 The number of hospital beds per capita


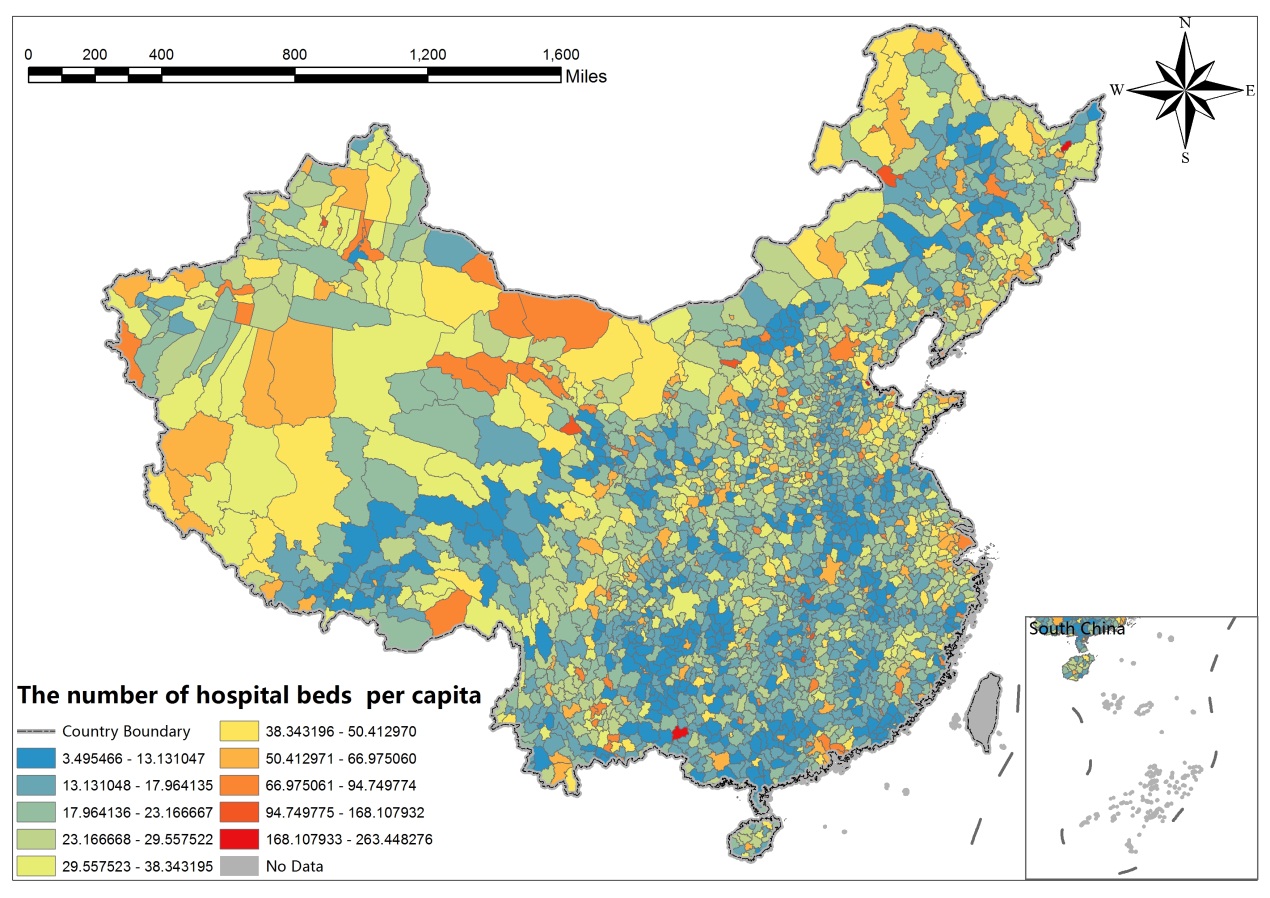


#### 2.2.4 The number of employees end of the year


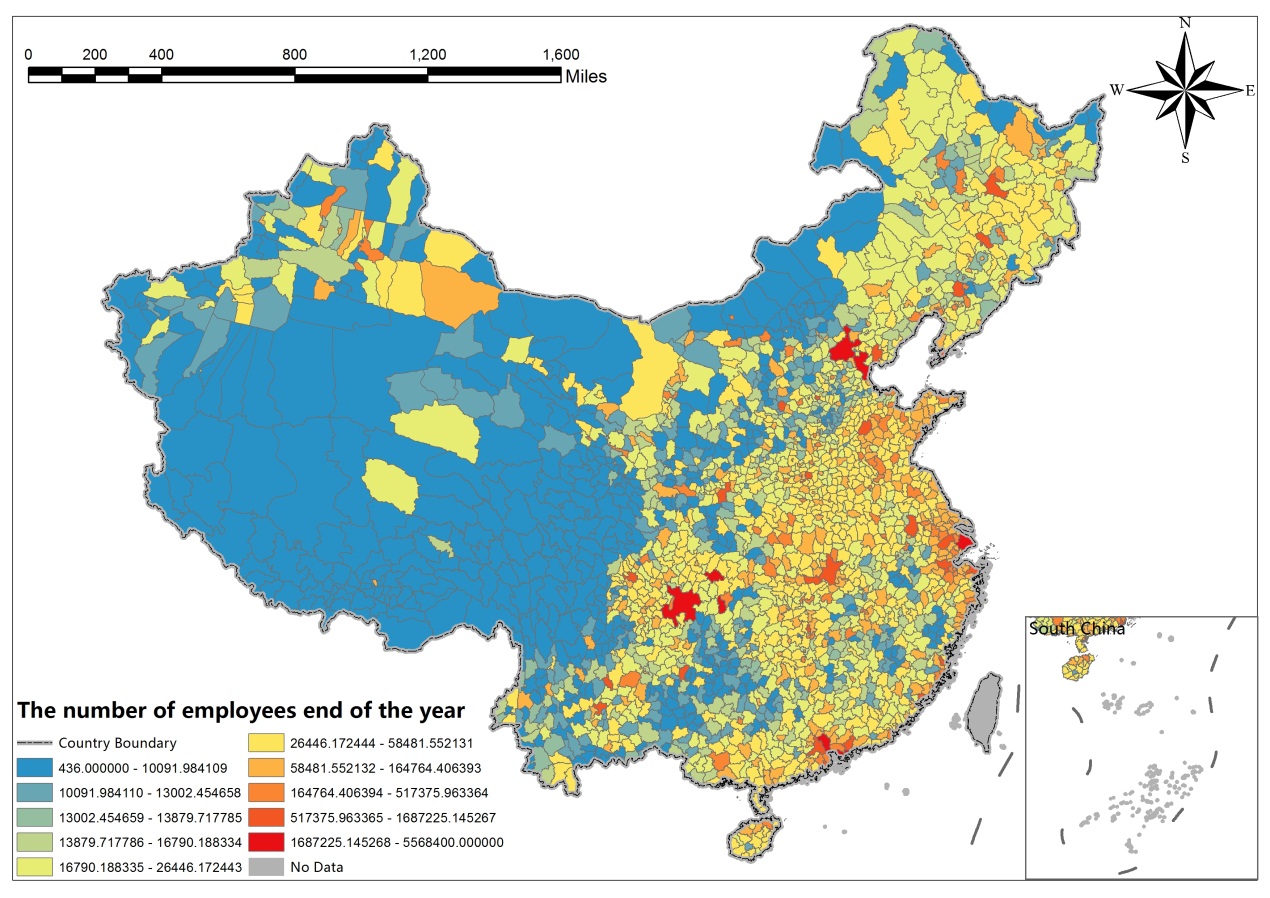


#### 2.2.5 Local general budget revenue (million)


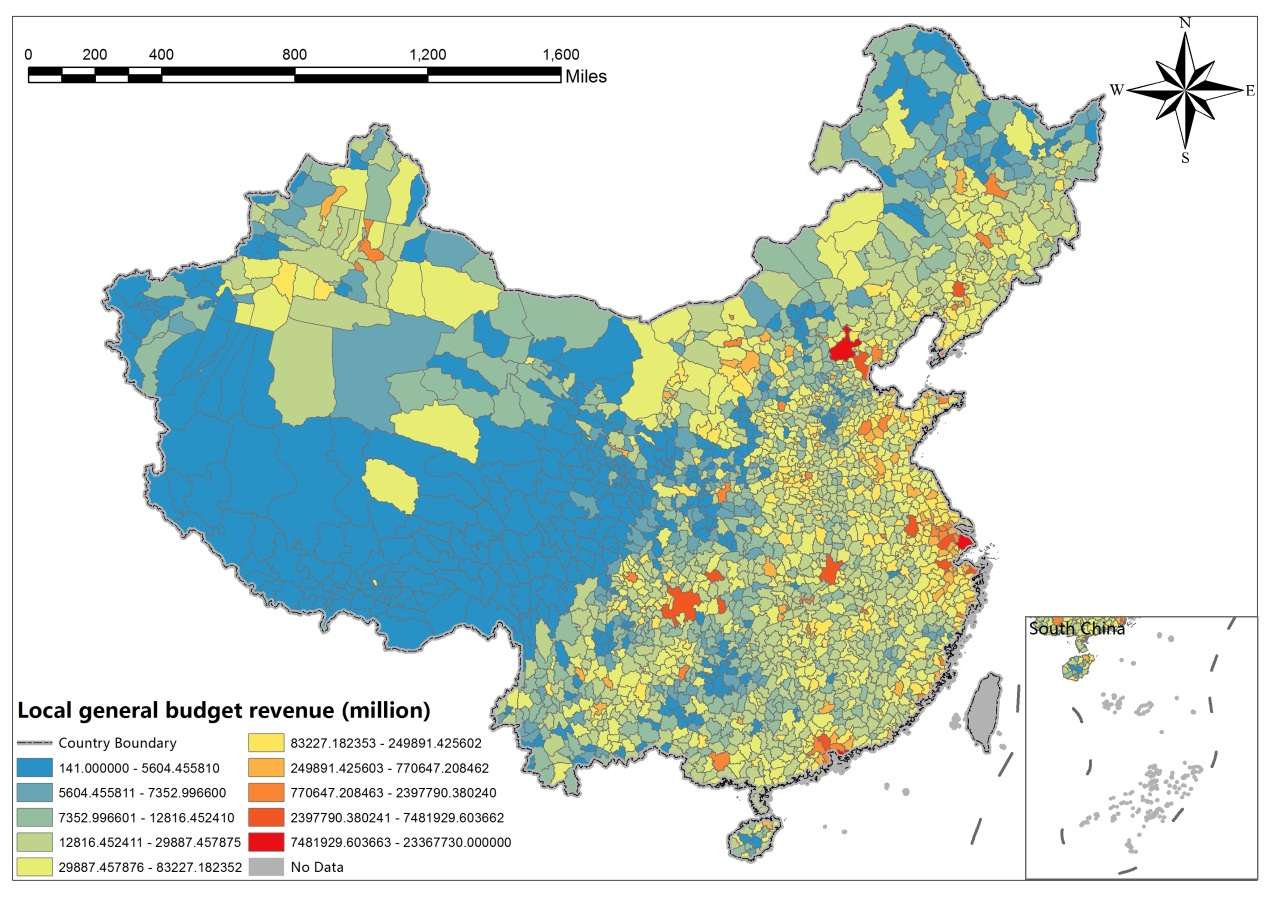


#### 2.2.6 Local Government Budgetary Expenditure (million)


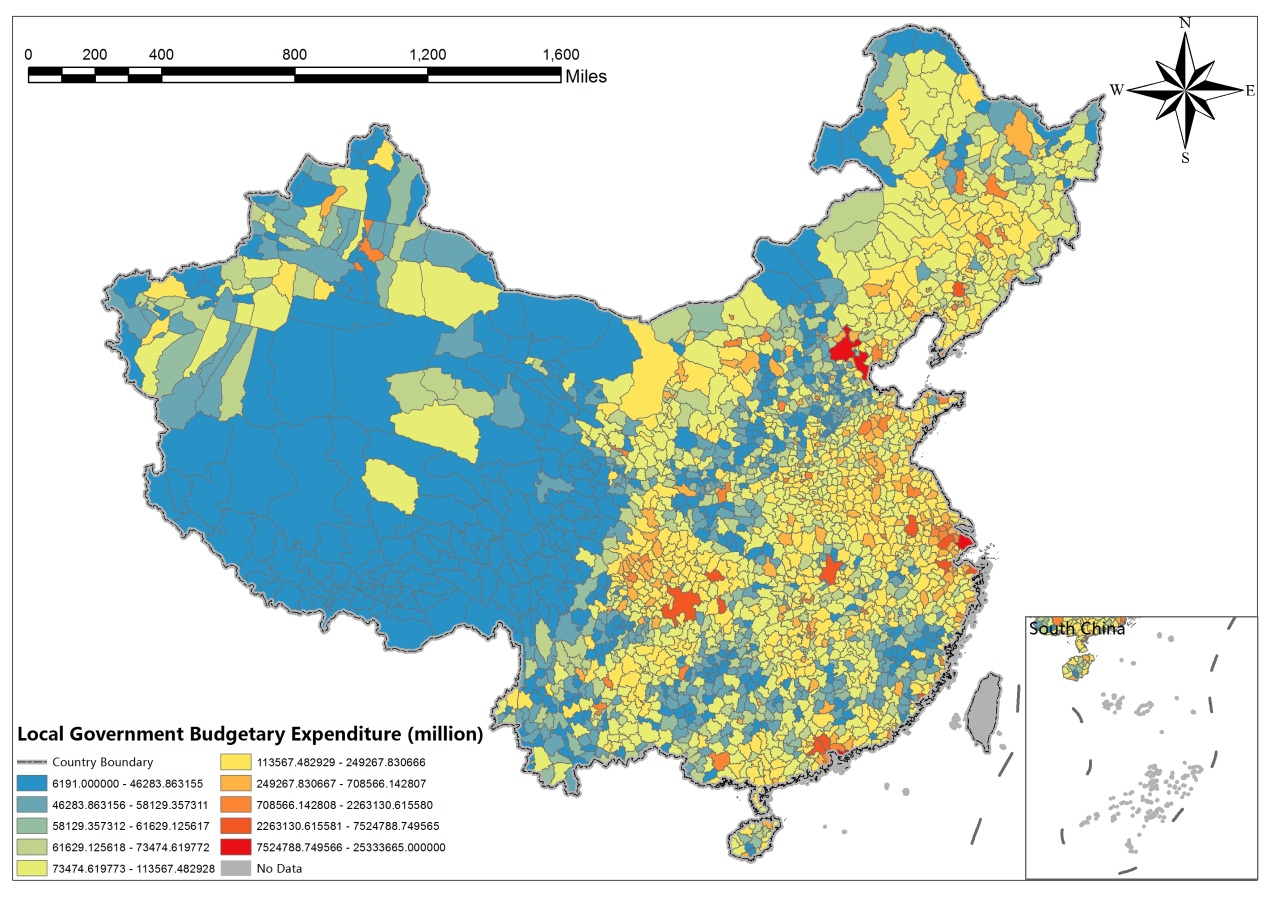


#### 2.2.7 Savings deposits of urban and rural residents (million)


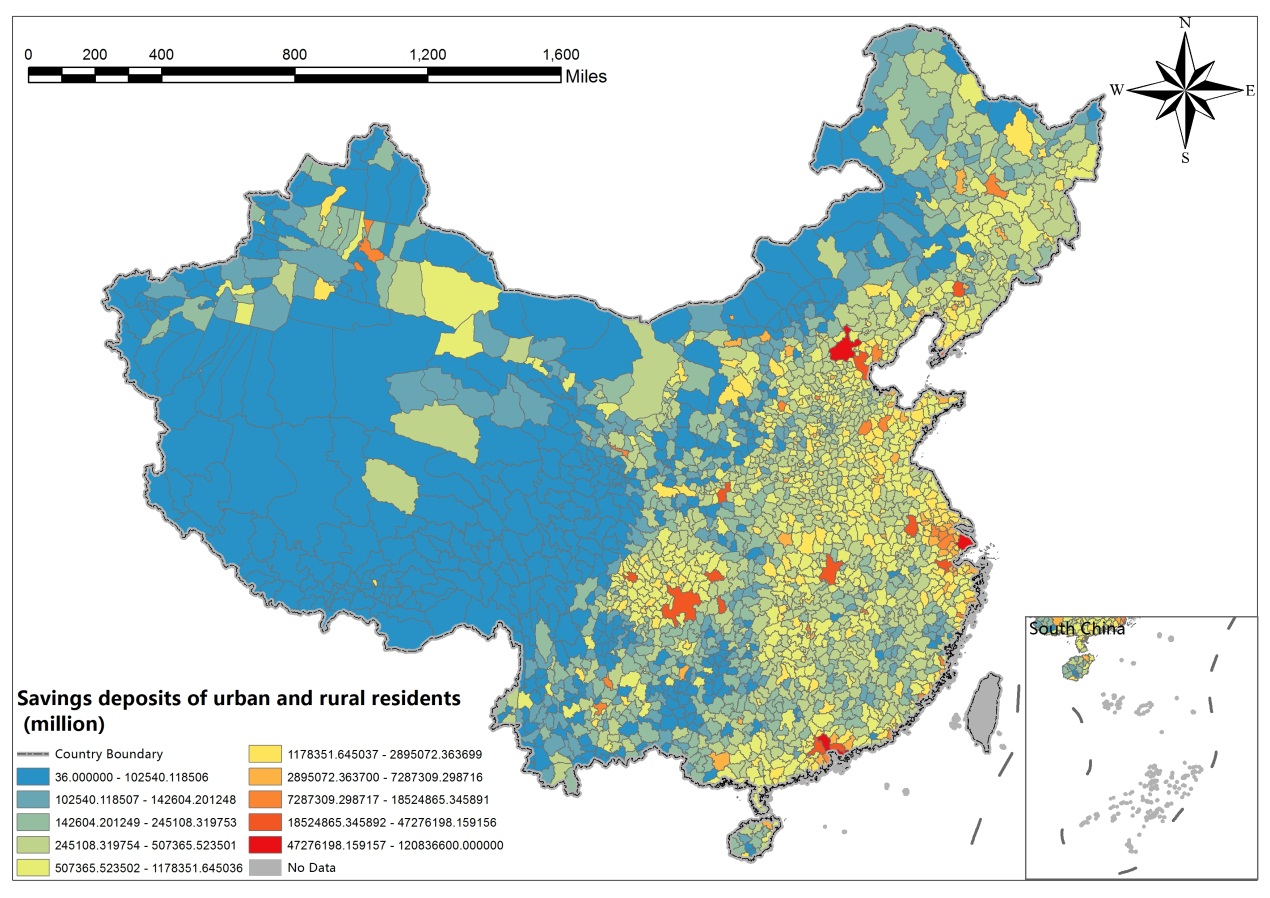


#### 2.2.8 The loan balance of the financial institutions end of the year (million)


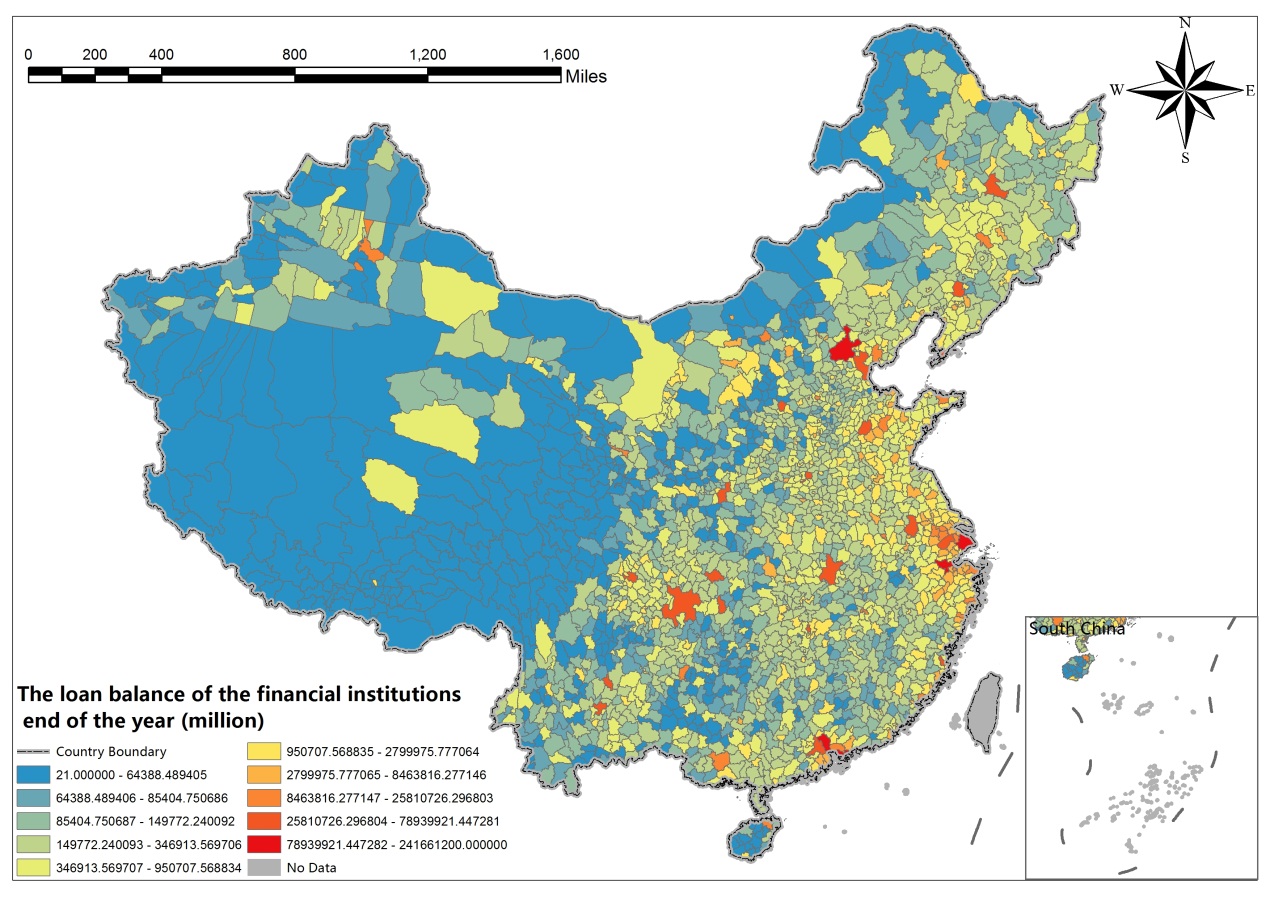


#### 2.2.9 The above-scale total industrial output value (million)


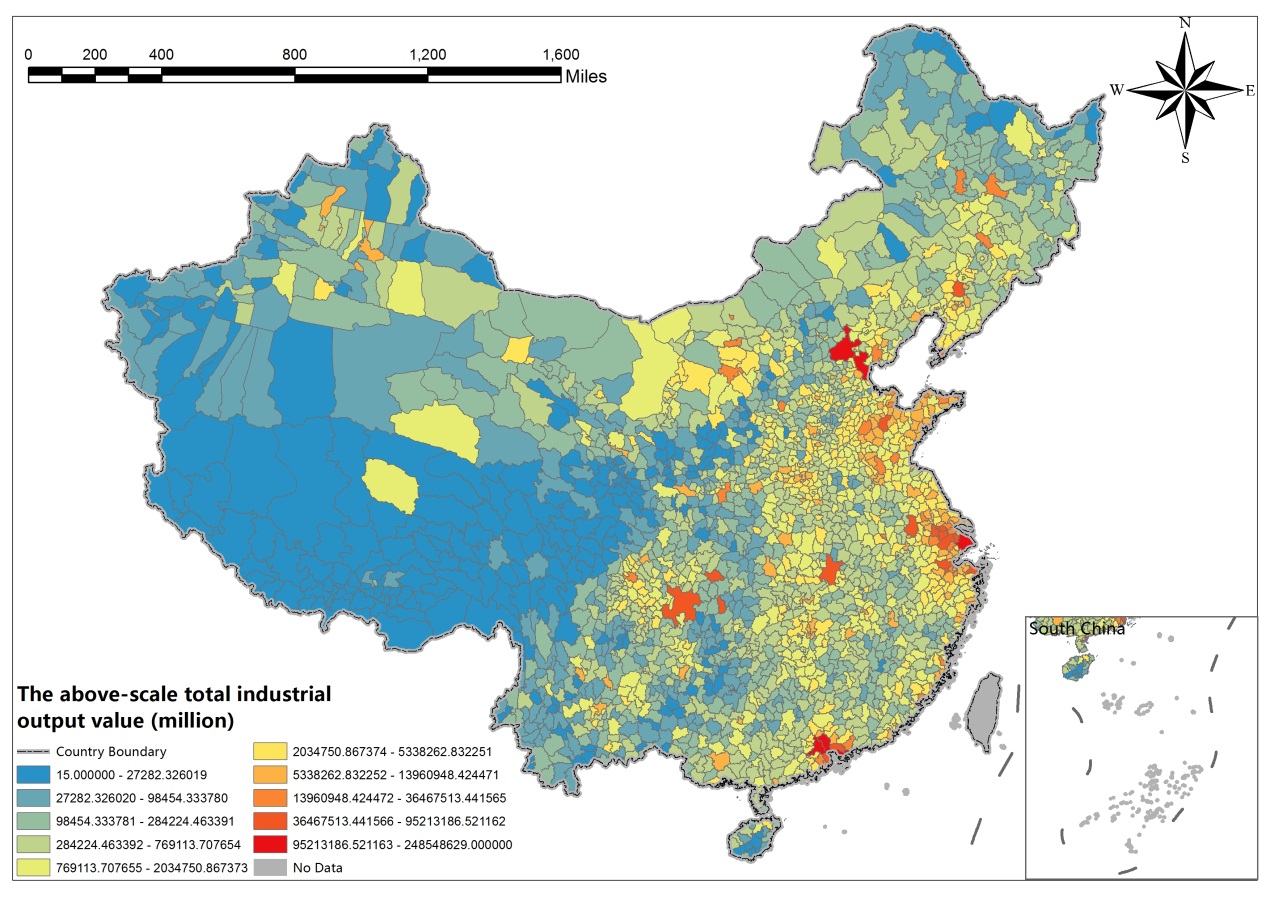


#### 2.2.10 Social fixed asset investment (million)


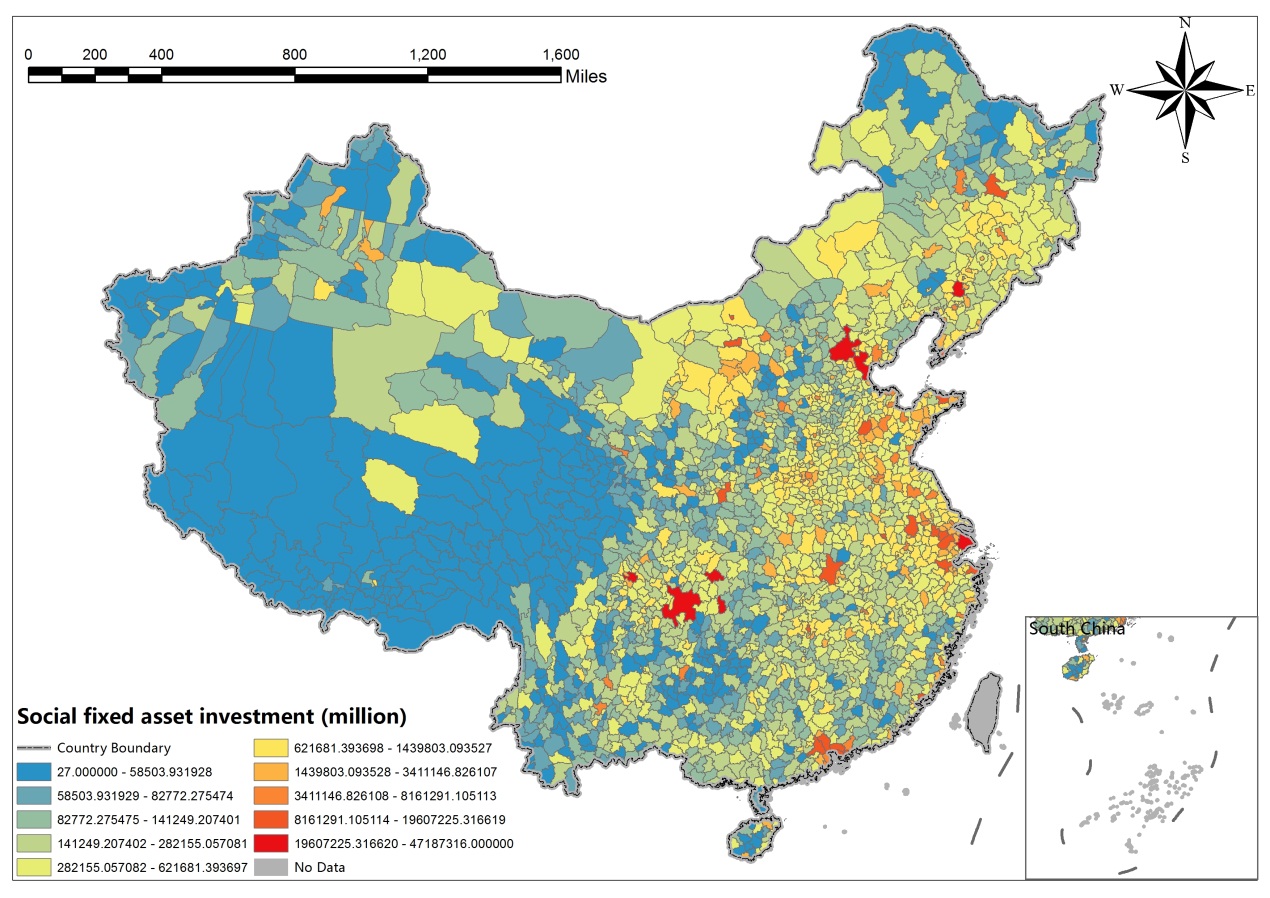


#### 2.2.11 General secondary school students (person)


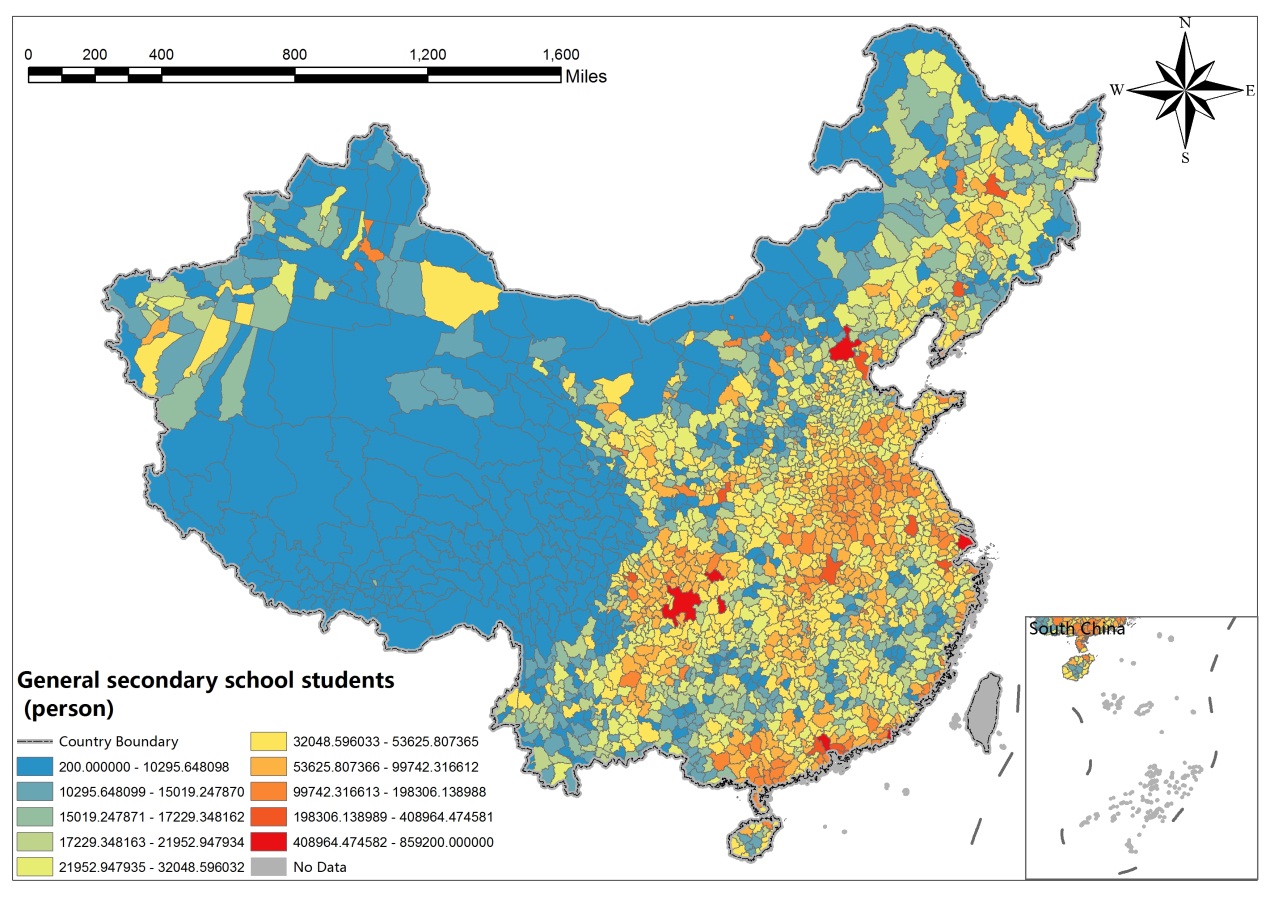


#### 2.2.12 Primary school students (person)


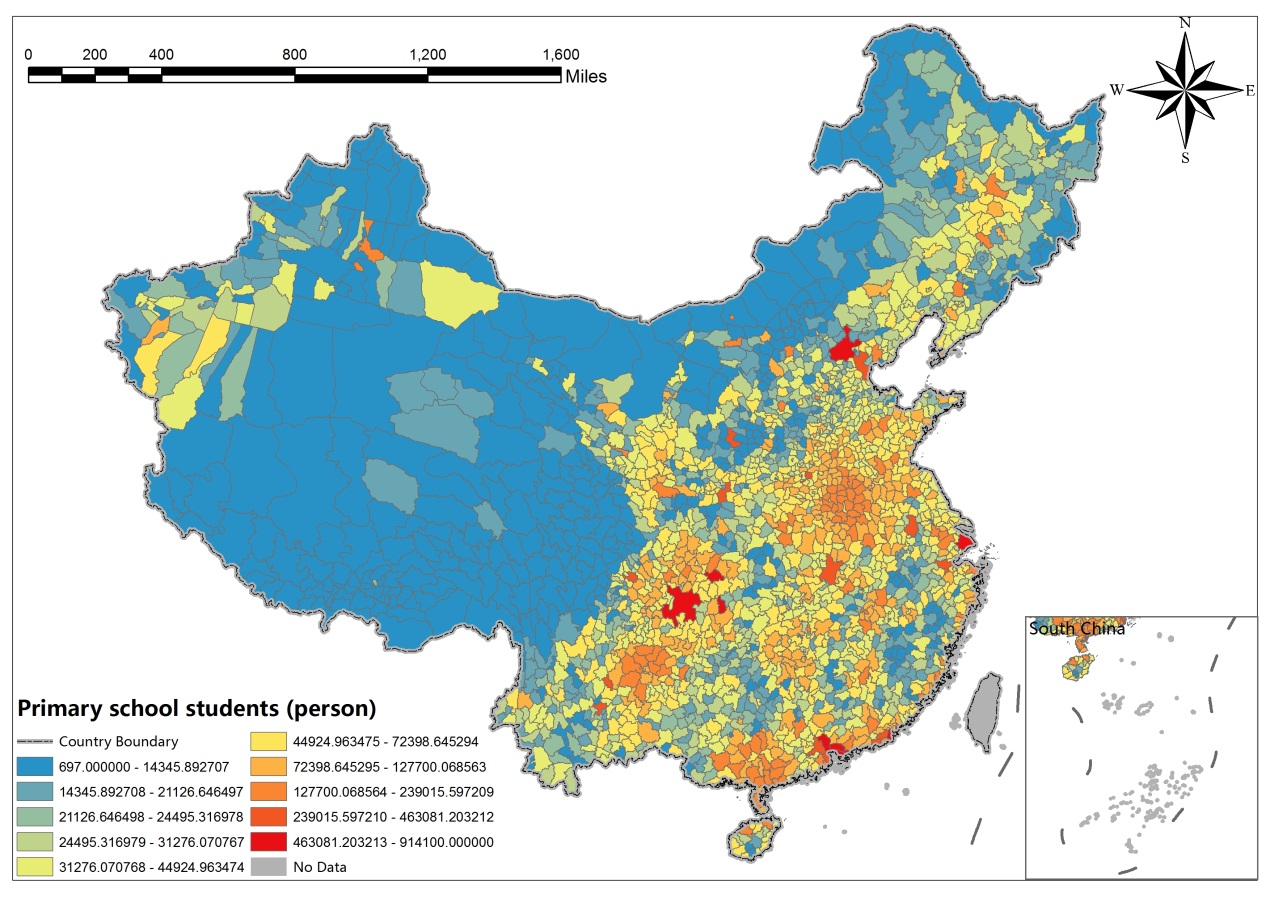


#### 2.2.13 Regional GDP (million)


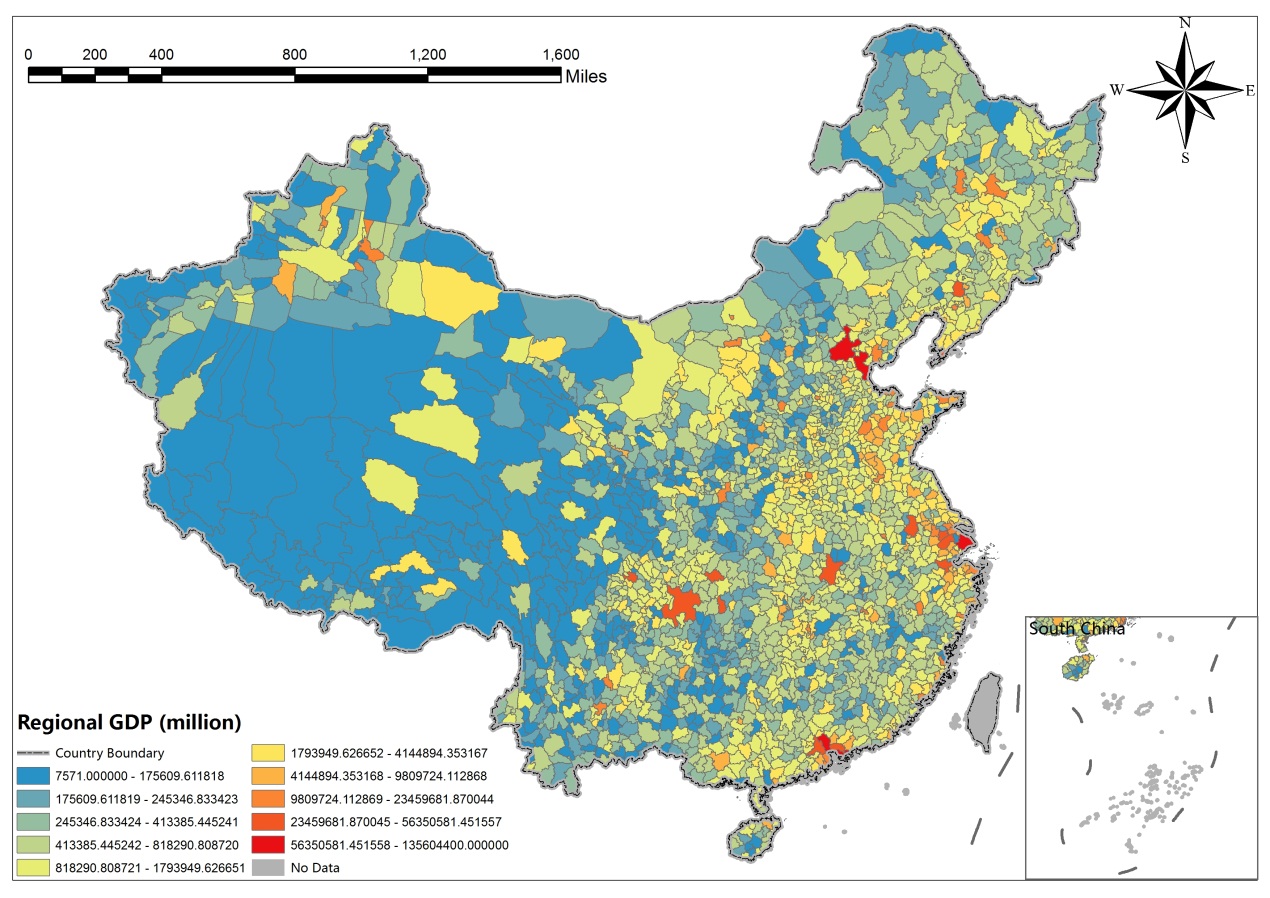


#### 2.2.14 The per capita GDP (Yuan / person)


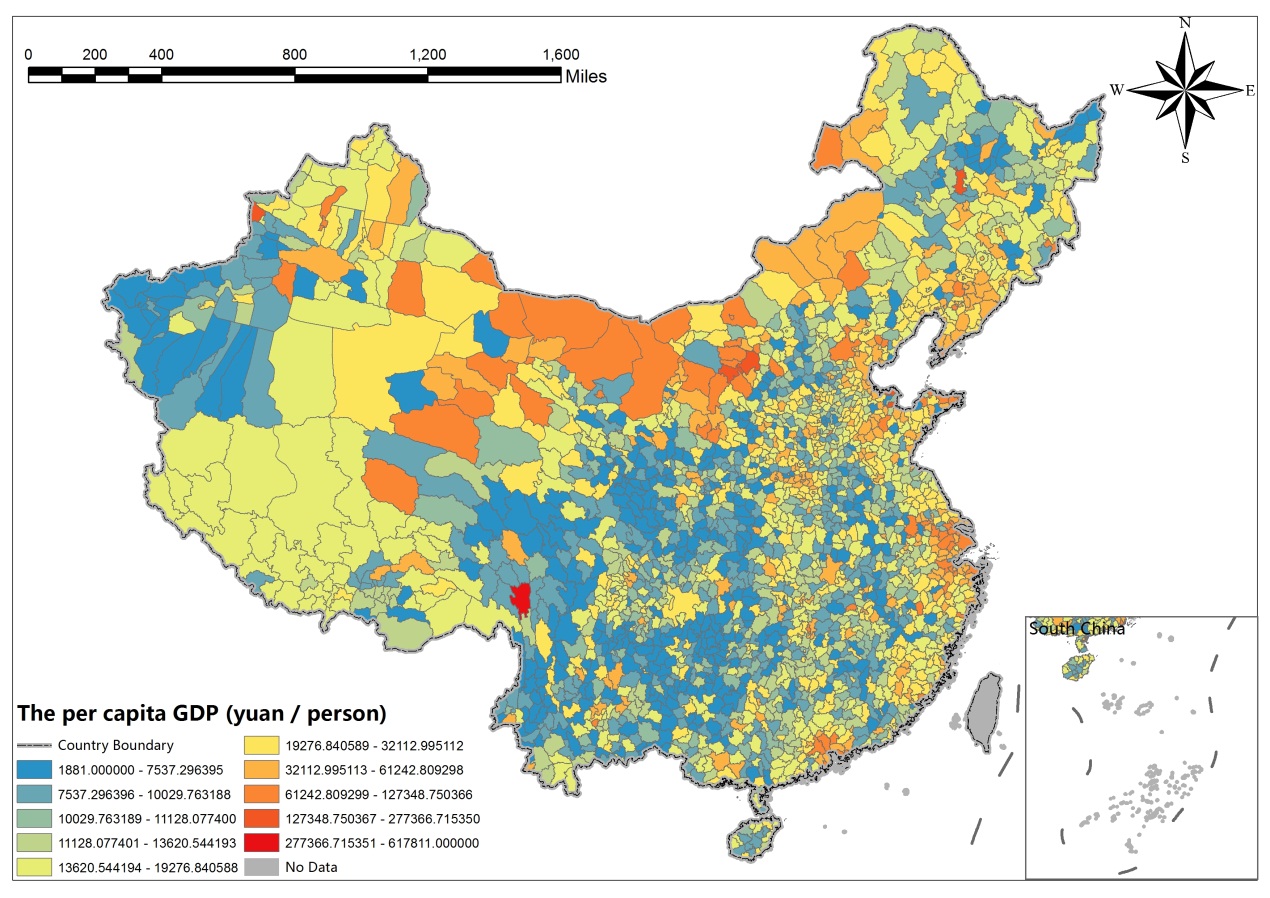


#### 2.2.15 The first industry output (million)


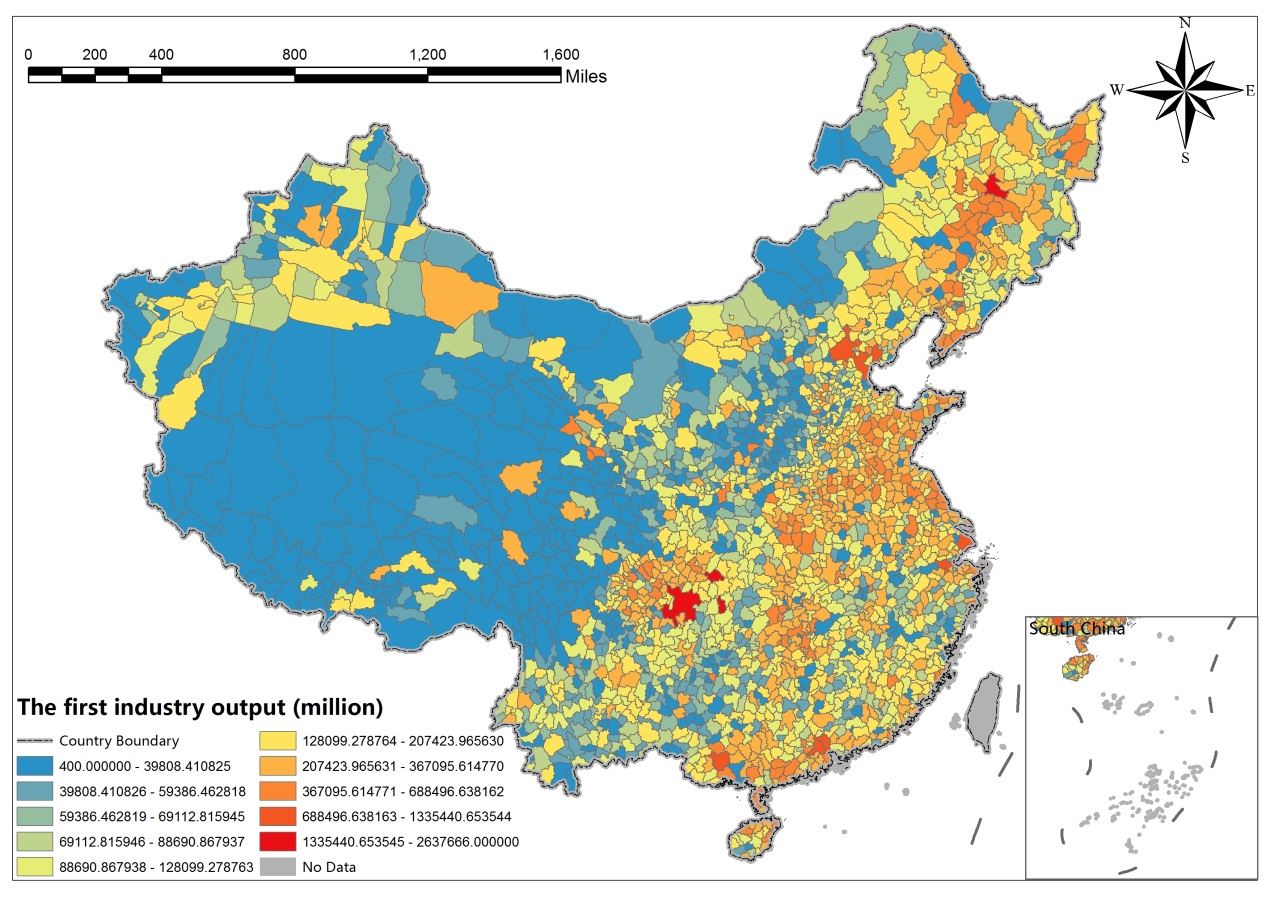


#### 2.2.16 The second industry output (million)


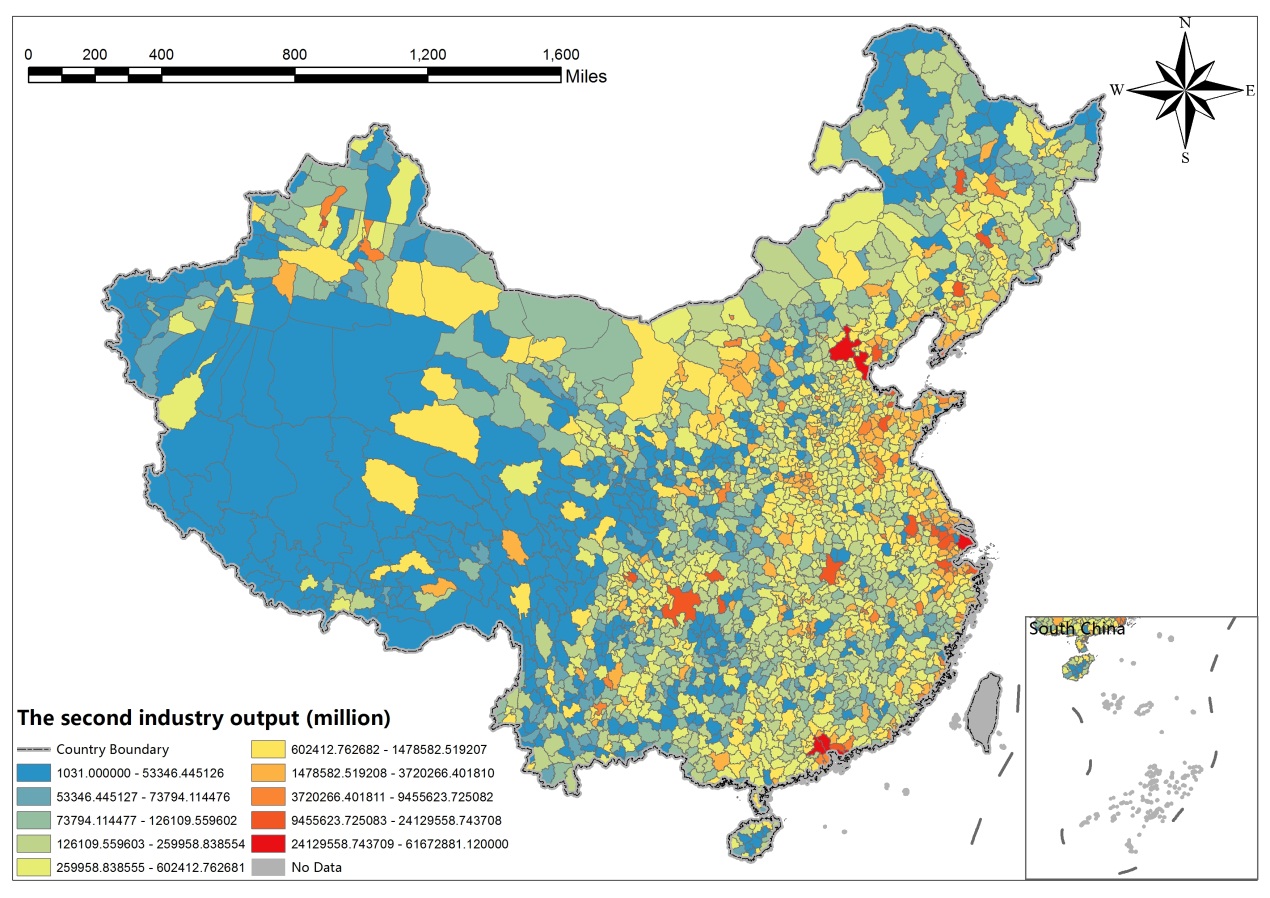


#### 2.2.17 Tertiary industry output value (million)


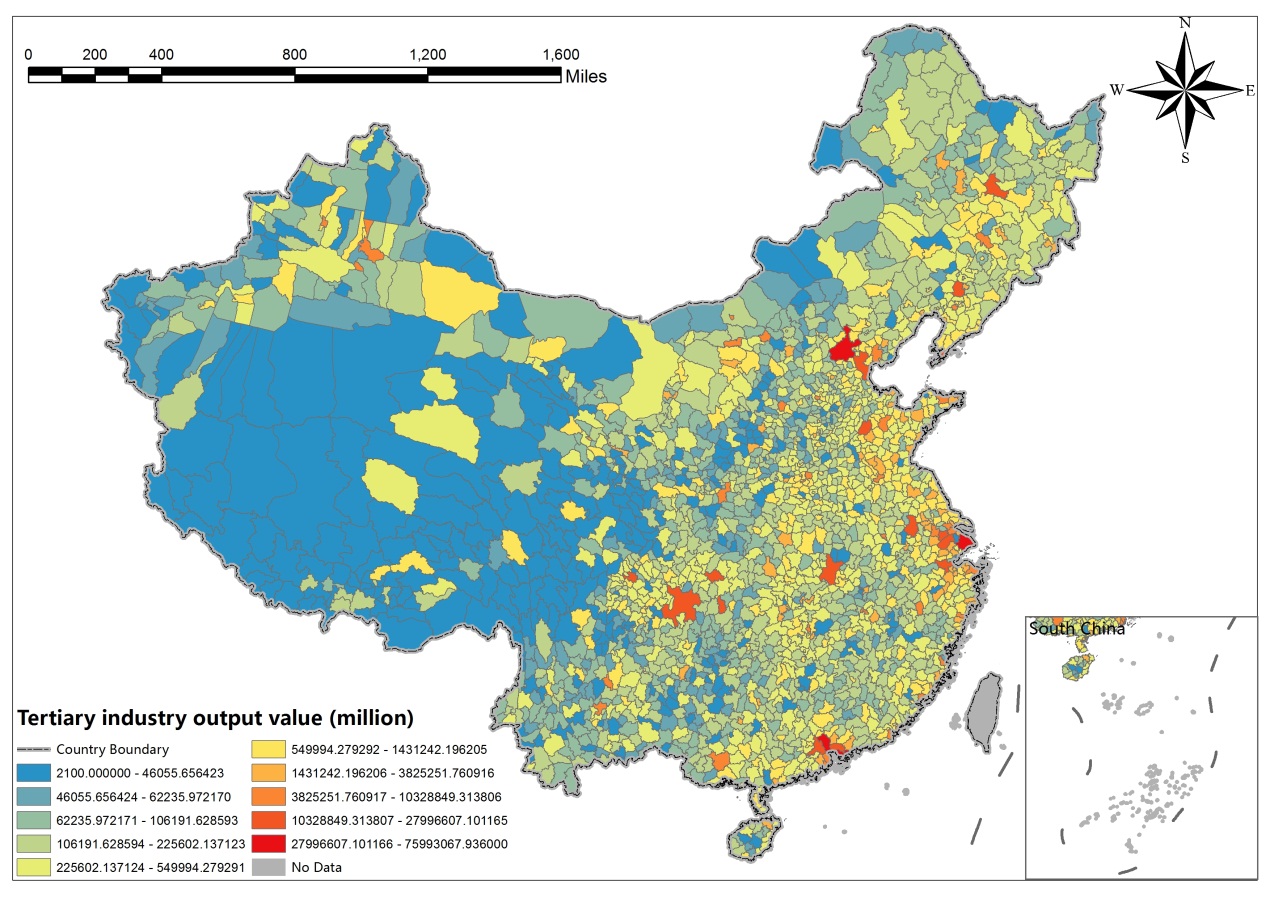


#### 2.2.18 Staff and Workers in Urban Units (person)


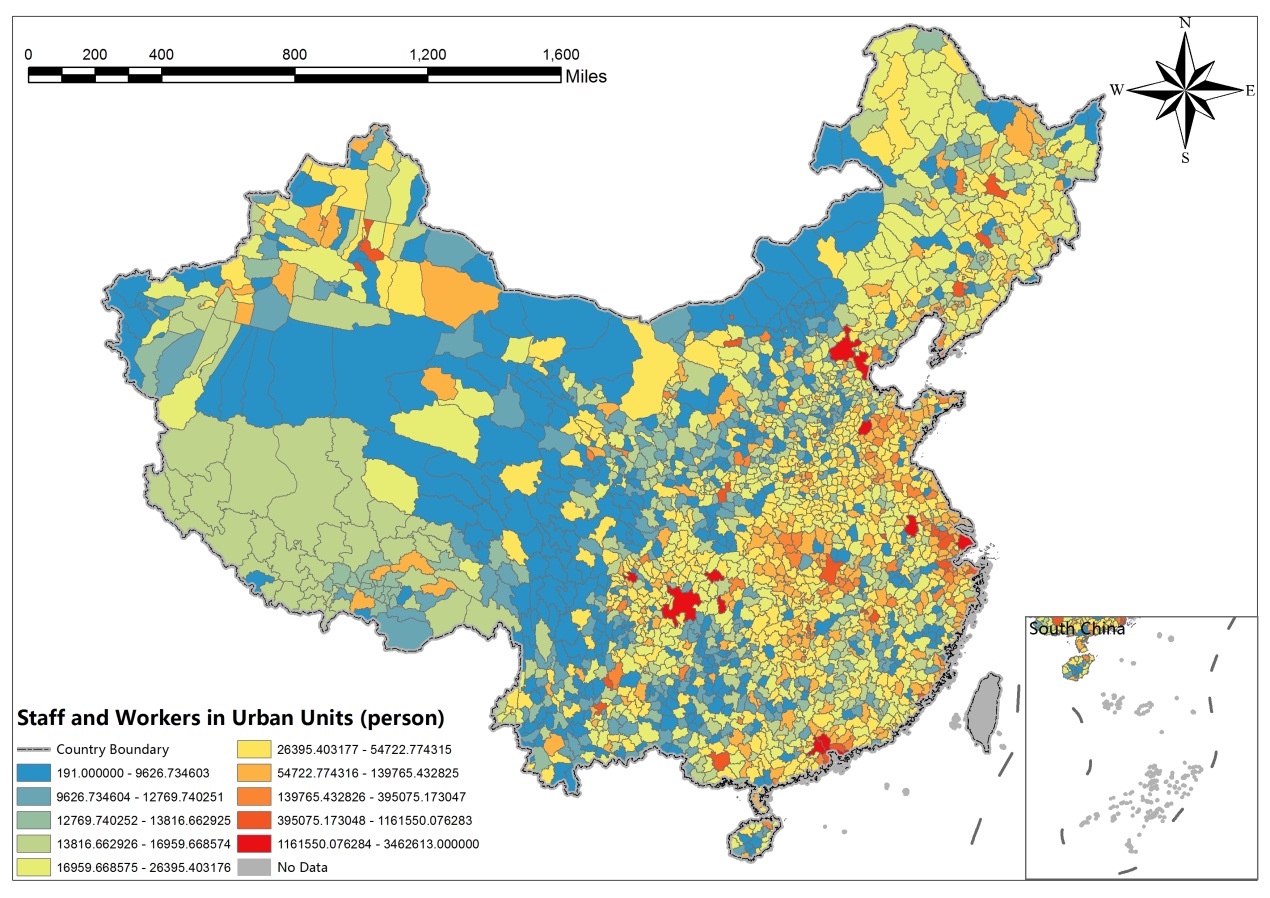


#### 2.2.19 Average wage of urban Staff and Workers (Yuan)


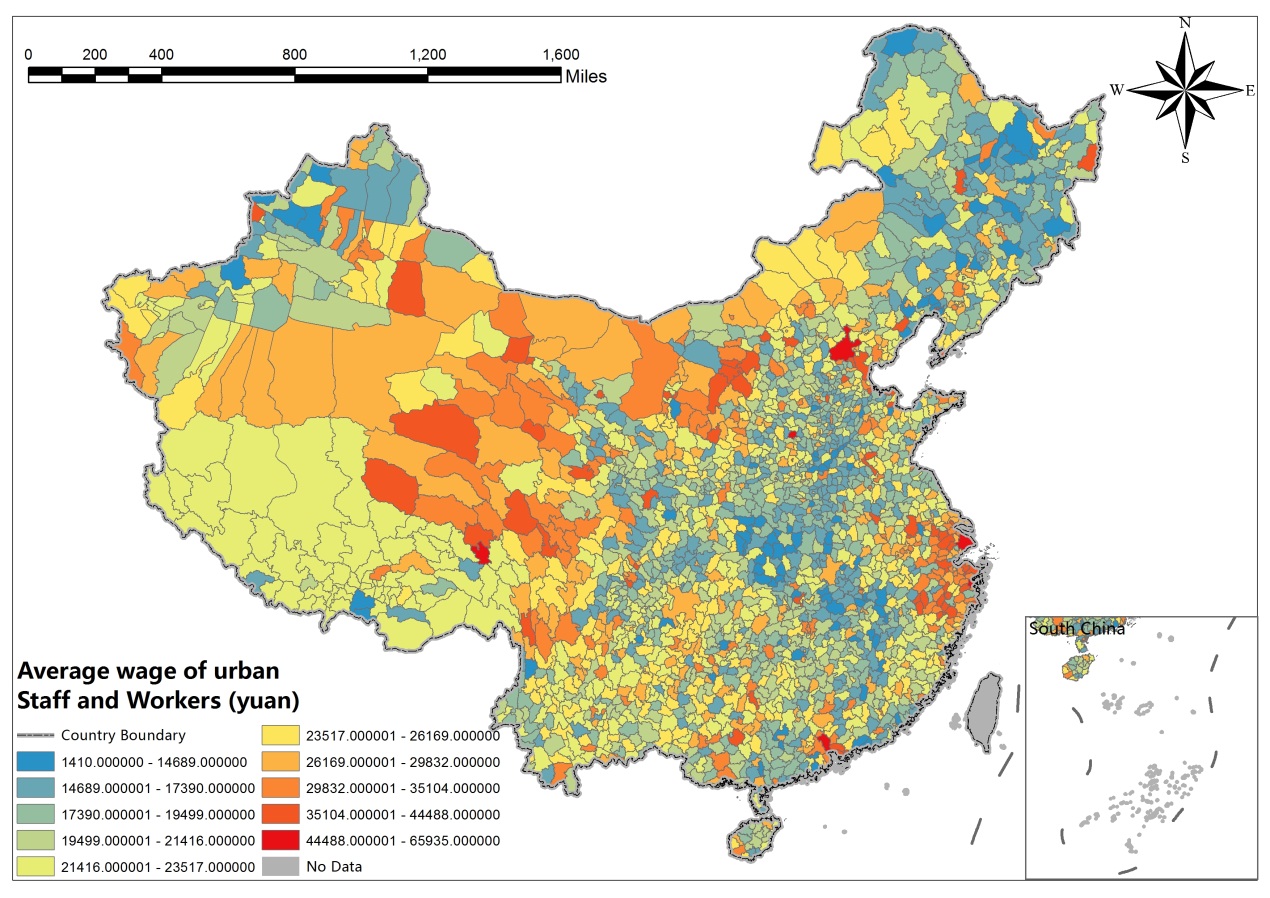


#### 2.2.20 The population density


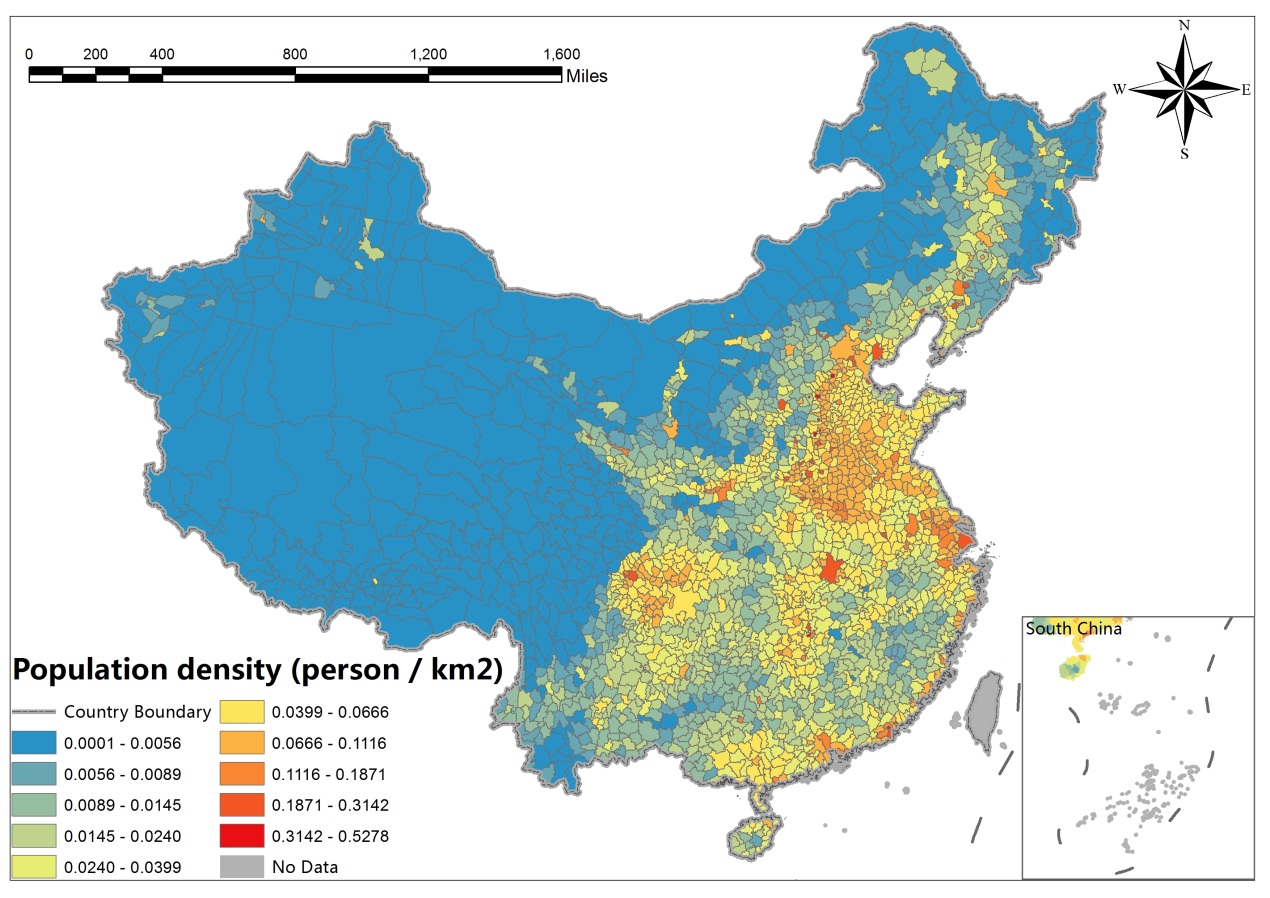


#### 2.2.21 The number of industrial enterprises above designated size


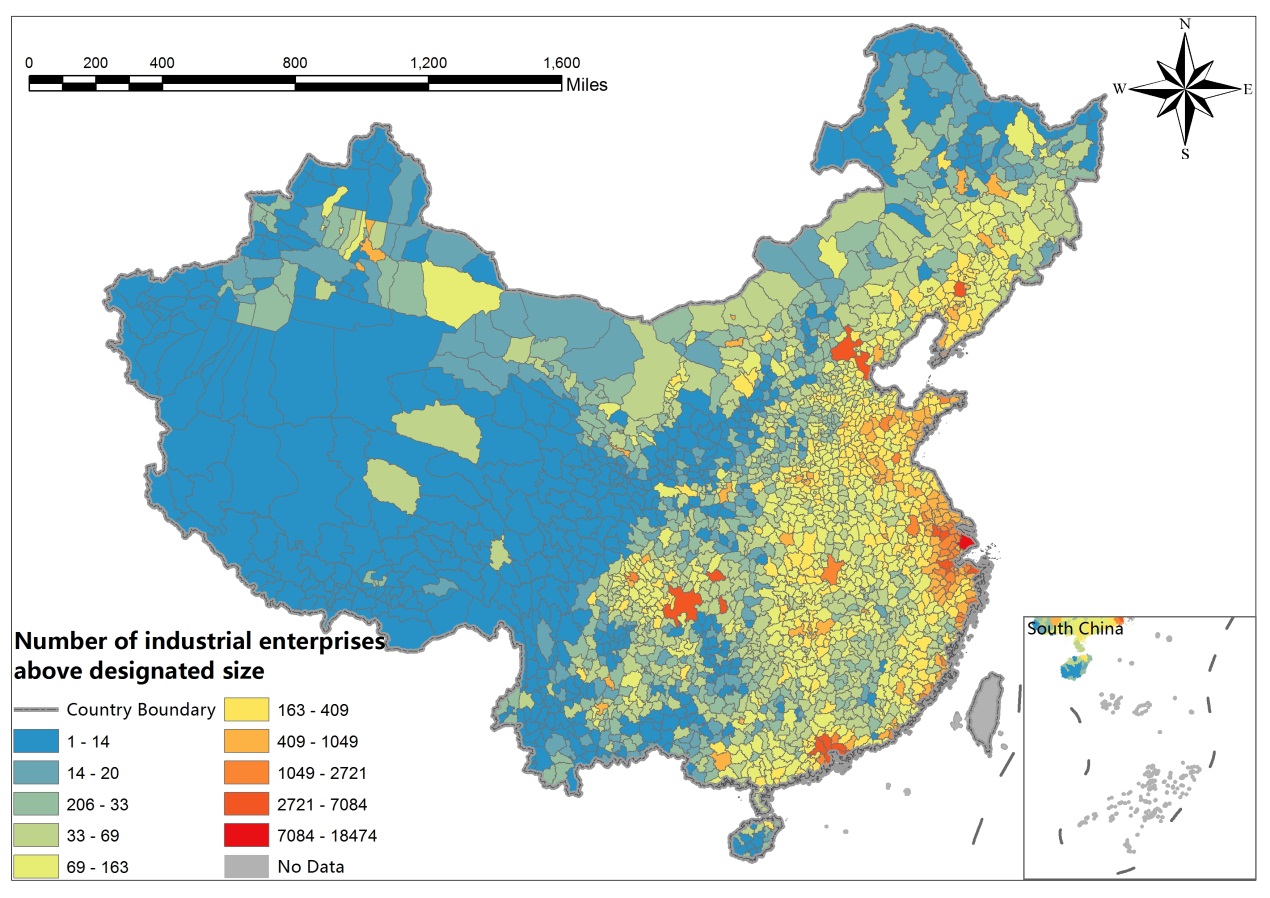


#### 2.2.22 The proportion of student population


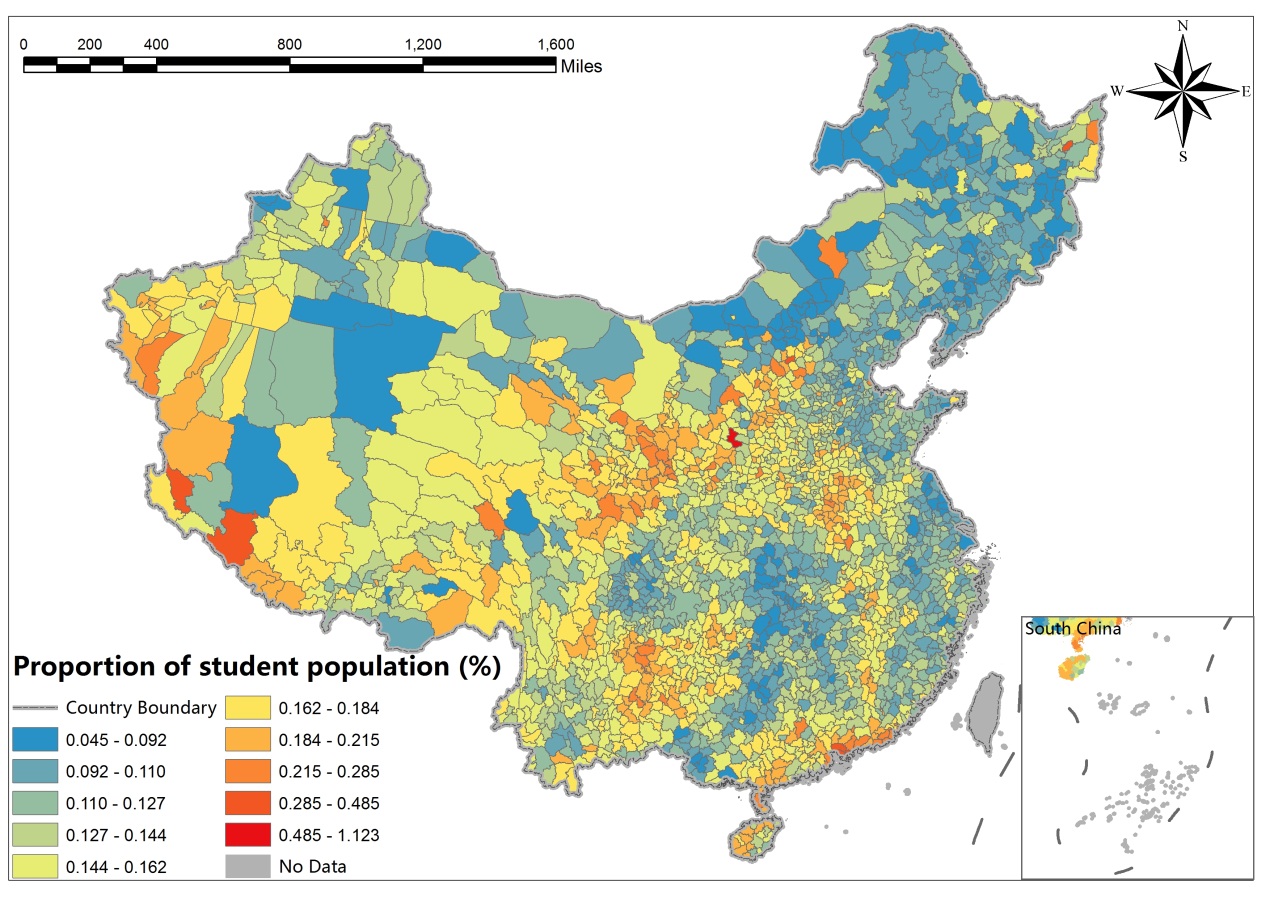

Supplement: Additional file 1 — The spatial distribution of the potential exposed variables. The Additional file 1 is standard Doc format. It introduces the spatial distribution of 29 potential exposed variables in this study, as reviewer suggested. [file 1471-2458-14-358-S1.docx]
